# Development of robust constitutive synthetic promoter using genetic resources of plant pararetroviruses

**DOI:** 10.3389/fpls.2024.1515921

**Published:** 2025-01-22

**Authors:** Tsheten Sherpa, Nrisingha Dey

**Affiliations:** ^1^ Division of Plant Biotechnology, Institute of Life Sciences, Bhubaneswar, India; ^2^ Regional Centre for Biotechnology, National Capital Region Biotech Science Cluster, Faridabad, India

**Keywords:** rational engineering, DNA shuffling, horseradish latent virus, mirabilis mosaic virus, figwort mosaic virus, as-1 element

## Abstract

With the advancement of plant synthetic biology, complex genetic engineering circuits are being developed, which require more diverse genetic regulatory elements (promoters) to operate. Constitutive promoters are widely used for such gene engineering projects, but the list of strong, constitutive plant promoters with strength surpassing the widely used promoter, the CaMV35S, is limited. In this work, we attempted to increase the constitutive promoter library by developing efficient synthetic promoters suitable for high-level gene expression. To do that, we selected three strong pararetroviral-based promoters from *Mirabilis mosaic virus* (MMV), *Figwort mosaic virus* (FMV), and *Horseradish latent virus* (HRLV) and rationally designed and combined their promoter elements. We then tested the newly developed promoters in *Nicotiana benthamiana* and found a highly active tri-hybrid promoter, MuasFuasH17 (MFH17). We further used these promoter elements in generating random mutant promoters by DNA shuffling techniques in an attempt to change/improve the MFH17 promoter. We further evaluated the activity of the MFH17 promoter in *Oryza sativa* seedlings and studied the effect of as-1 elements present in it. Finally, we tested the efficacy and tissue specificity of the MFH17 promoter *in planta* by developing transgenic *Nicotiana tabacum* and *Arabidopsis thaliana* plants and found it highly constitutive and efficient in driving the gene throughout the plant tissues. Overall, we conclude that this tripartite synthetic promoter MFH17 is a strong, highly constitutive, and dual-species (dicot and monocot) expressing promoter, which can be a valuable addition to the constitutive plant promoter library for plant synthetic biology.

## Introduction

Synthetic biology is building new properties into living systems by combining engineering, DNA technology, and computer science to make something useful (Davies, 2019; Hanson et al., 2019). Advancement of synthetic biology in plants holds high importance, as it has vast applications such as the production of value-added metabolites through metabolic engineering (Liu et al., 2023; Selma et al., 2023), biosensor development (Adams et al., 2012; Walia et al., 2018), bioremediation of environmental toxins (Rylott and Bruce, 2020; Aminian-Dehkordi et al., 2023), development of bio-fortified crops (Van Der Straeten et al., 2020; Edwards et al., 2024) and developing stress-resistant plants (Foo et al., 2018; Yang et al., 2021). However, plant synthetic biology still lags behind the microbial field because of higher genomic complexity, longer life cycles of plants, and since this field in plants is comparatively younger and, therefore, has limited available genetic tools (Patron, 2020; Tian et al., 2022; Joshi and Hanson, 2024).

One of the most important modules in any synthetic biology toolkit is a promoter responsible for expressing and regulating the gene of interest. A plant promoter comprises three important regions: a core promoter region where RNA polymerase binds and initiates the transcription; a proximal promoter region, where different transcription factors bind to the cis-regulatory elements (CRE) and regulate the promoter activity; and a distal promoter region which is usually present far away from the core promoter region and regulates the promoter activity. The difference in the regulatory sequence in these regions leads to different types of promoters, such as constitutive promoters, which express the gene in all tissues at all times, a spatial/temporal specific promoters, which express the gene only in specific tissues or in specific developmental stage and an inducible promoters where gene expression gets induced by certain chemicals or molecules (Peremarti et al., 2010). Though all these types of promoters are equally important in synthetic biology applications, constitutive promoters have seen the widest use because of their significance in metabolic engineering projects for essential metabolites/proteins, developing synthetic genetic circuits, and generating herbicide-tolerant plants, which requires high gene expression and ubiquitous expression in all tissues (Liu and Stewart, 2016; Mallah et al., 2017). However, the number of well-characterized strong constitutive plant promoters whose activity surpasses the commonly used constitutive promoter, the 35S, and with dual-species (monocot and dicot) expression capability is low, and the continuous use of the same set of promoters in increasingly complex genetic engineering circuits can lead to homology-based gene silencing (HBGS) causing impairment of transgene expression (Dietz-Pfeilstetter, 2010; Rajeevkumar et al., 2015). Therefore, developing more well-characterized, strong constitutive plant promoters is crucial.

Plant pararetrovirus, a double-stranded DNA, plant infecting viruses, has evolved strong promoters for its propagation in its host plants. Widely known constitutive promoters such as 35S or 2X35S from *Cauliflower mosaic virus* [CaMV; Kay et al. (1987)], M12 and M24 from *Mirabilis mosaic virus* [MMV; Dey and Maiti (1999); Sahoo et al. (2014)] P-CsVMV from *Cassava vein mosaic virus* [CsVMV; Verdaguer et al. (1998); Deb et al. (2018)] and P-FMV from *Figwort mosaic virus* [FMV; Maiti et al. (1997)] are all pararetroviral-based and are highly efficient in driving transgene in both transient and transgenic plant system. We previously characterized a strong, constitutive, and multi-stress inducible pararetroviral promoter from *Horseradish latent virus* (HRLV) full-length transcript promoter, the H17 (short fragment) and H12 (long fragment) promoter, which showed efficient gene expression in transgenic *Arabidopsis thaliana* and *Nicotiana tabacum* plants (Khan et al., 2018). Therefore, these viruses are a treasure trove of useful genetic elements for developing strong, constitutive synthetic plant promoters.

This study aimed to develop strong, constitutive plant promoters, which will be useful in any synthetic biology application requiring high-level gene expression and protein production. We selected three strong pararetroviral-based full-length transcript promoters from *Mirabilis mosaic virus* (MMV), *Figwort mosaic virus* (FMV), and *Horseradish latent virus* (HRLV) and rationally designed and fused the promoter elements in different combinations; subsequently, we tested all the promoters in *Nicotiana benthamiana* and identified a highly active recombinant promoter, MuasFuasH17 (MFH17), consisting of a combination of above three promoter elements. We further attempted to improve the activity of the MFH17 promoter using DNaseI-based and oligos-based *in vitro* DNA shuffling methods. We also checked the activity of the MFH17 promoter in rice seedlings and analyzed its efficiency in driving the *GFP* reporter gene. The effect of as-1 elements on MFH17 promoter activity was also evaluated. Furthermore, the expression and tissue specificity of MFH17 were studied in both *Nicotiana tabacum* and *Arabidopsis thaliana* transgenic plants. Finally, we proclaim that MFH17 developed in this study could become an important genetic tool in plant synthetic biology.

## Materials and methods

### Materials

All genetic materials, such as *Horseradish latent virus* (HRLV)*, Mirabilis mosaic virus* (MMV)*, Figwort mosaic virus* (FMV), and *Cauliflower mosaic virus* (CaMV) promoter elements and seeds of *Nicotiana tabacum* and *Nicotiana benthamiana* were kindly offered by Dr. I. B. Maiti, University of Kentucky, USA. All oligonucleotides were synthesized from Eurofins Scientific, Luxembourg, and all enzymes used in this study, such as T4 DNA ligase, PNK, restriction enzymes, *Taq* DNA Polymerase, and DNaseI were procured from Thermo Fisher Scientific Inc. (Waltham, MA). Chemicals such as X-Gluc, DEPC, Agarose, 4-methylumbelliferyl-beta-D-glucuronide (MUG), etc., were purchased from Sigma-Aldrich (St. Louis, USA). The SYBR green master mix (GoTaq^®^ qPCR) was bought from Promega Corporation, Madison, Wisconsin (Cat. No. A6001), the First Strand cDNA Synthesis Kit from Thermo Fisher Scientific Inc. (Cat. No. K1622), and the RNeasy Plant Mini Kit from QIAGEN (Cat. No. 74904), Hilden, Germany. The basic chemicals such as Tris, agar, Luria Broth (LB), Murashige and Skoog (MS) medium media, MES, KCL, MgCl_2_, acetosyringone, Kanamycin, Rifampicin, etc., were obtained from HiMedia Laboratories, from Mumbai, India.

### Construction of plasmids

#### Development of hybrid promoter constructs

**Figure 1:**
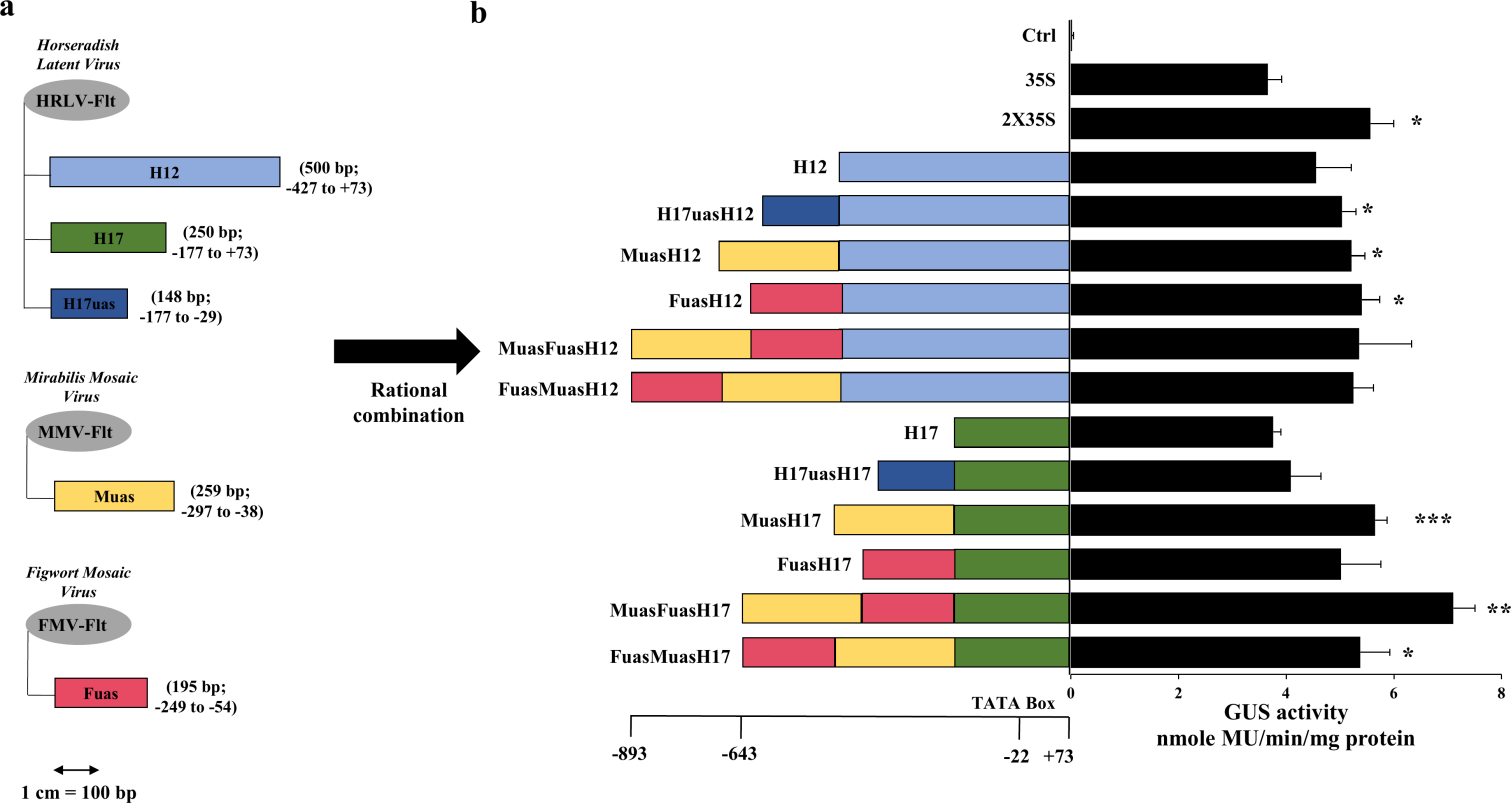
Rational design of hybrid synthetic promoters. **(A)** The full-length transcript (Flt) promoter from *Mirabilis mosaic virus* (MMV-Flt), *Figwort mosaic virus* (FMV-Flt), and *Horseradish latent virus* (HRLV-Flt) was used as parent promoter in generating five promoter fragments, namely Fuas (195 bp) from FMV, Muas (259 bp) from MMV and H17uas (148 bp), H17 (250 bp) and H12 (500 bp) from HRLV, where Fuas, Muas, and H17uas were used as upstream activating sequence, and H17 and H12 was used as core promoter. **(B)** The five promoter fragments were used to develop ten hybrid promoters, namely H17uasH12 (654 bp), MuasH12 (765 bp), FuasH12 (701 bp), MuasFuasH12 (966 bp), FuasMuasH12 (966 bp), H17uasH17 (404 bp), MuasH17 (515 bp), FuasH17 (451 bp), MuasFuasH17 (716 bp), FuasMuasH17 (716 bp) and their transient promoter activity analysis was done in *N. benthamiana* along with Ctrl (VC), H12, H17, 35S, and 2X35S promoters. Their average GUS activity, respective SD, and statistical significance (*p ≤ 0.05; **p ≤ 0.01; ***p ≤ 0.001) are represented.

A total of five promoter fragments was used for hybrid development where three fragments were generated from a *Horseradish latent virus* full-length transcript (HRLV-Flt) promoter [Khan et al. (2018); NCBI Reference Sequence: NC_018858.1], named as H12 (500 bp; -427 to +73), H17 (250 bp; -177 to +73) and H17uas (148 bp; -177 to -29), one fragment from *Mirabilis mosaic virus* full-length transcript (MMV-Flt) promoter (NCBI Reference Sequence: NC_004036.1), named, Muas [259 bp, -297 to -38; Dey and Maiti (1999)] and one fragment from *Figwort mosaic virus* full-length transcript (FMV-Flt) promoter (NCBI Reference Sequence: NC_003554.1), named Fuas [195 bp, -249 to -54; Maiti et al. (1997)]. These fragments were first PCR amplified with primers having EcoRI (GAATTC) and HincII (GTCGAC) restriction sites as 5’ overhang and SmaI (CCCGGG) and HindIII (AAGCTT) restriction sites as 3’ overhang and cloned into pUC119 vector, generating five clones, namely pUCH12, pUCH17, pUCH17uas, pUCMuas and pUCFuas. These clones were then hybridized following the previously published protocol described in Kumari et al. (2024), where the H17 and H12 fragments were kept as a core promoter fragment, and the H17uas, Muas, and Fuas were hybridized upstream of these two core promoters in different combinations (**Figure 1A**). The resulting hybrid promoters were individually cloned in a plant expression vector pKYLXGUS vector (Schardl et al., 1987), along with 35S, 2X35S, H12 and H17 promoters resulting in vectors, pLXH17uasH12GUS, pLXMuasH12GUS, pLXFuasH12GUS, pLXMuasFuasH12GUS, pLXFuasMuasH12GUS, pLXH17uasH17GUS, pLXMuasH17GUS, pLXFuasH17GUS, pLXMuasFuasH17GUS, pLXFuasMuasH17GUS, pLX35SGUS, pLX2X35SGUS, pLXH12GUS and pLXH17GUS and the resultant plasmids were named H17uasH12, MuasH12, FuasH12, MuasFuasH12, FuasMuasH12, H17uasH17, MuasH17, FuasH17, MuasFuasH17 (in short MFH17), FuasMuasH17, 35S, 2X35S, H12 and H17, respectively (**Figure 1B**).

The sequences of the corresponding promoters, along with their sizes, can be found in **Supplementary Data 1** in **Supplementary Data Sheet 1**. The primer sequences are available in **Supplementary Data 2** in **Supplementary Data Sheet 1**, and the gel images of the restriction digestion of rationally designed hybrid promoter construct cloned into pUC119 and pKYLXGUS vectors using EcoRI and HindIII are included in **Supplementary Data 3** in **Supplementary Data Sheet 1**.

Furthermore, in the vectors pLXMuasFuasH17GUS, pLX35SGUS, and pLX2X35SGUS, the *GUS* gene was replaced by the *GFP* gene, leading to the development of pLXMuasFuasH17GFP, pLX35SGFP and pLX2X35SGFP vectors.

#### Development of as-1 element mutant promoter constructs

Previously published PCR-based site-directed mutation was performed for mutation of as-1 element pairs in MuasFuasH17 (in short MFH17) promoter (Reikofski and Tao, 1992). A total of seven mutant promoters were developed, namely, M (as-1 pair mutation in Muas), F (as-1 pair mutation in Fuas), H (as-1 pair mutation in H17), MF (as-1 pair mutations in Muas and Fuas), FH (as-1 pair mutations in Fuas and H17), MH (as-1 pair mutations in Muas and H17) and MFH (as-1 pair mutations in Muas, Fuas and H17) using modified primers followed by overlap-extension PCR (**Supplementary Data 2** in **Supplementary Data Sheet 1**). The mutant promoters created were subsequently inserted into the pKYLXGUS vector for additional analysis.

### DNA shuffling by DNaseI enzyme

The hybrid promoter MuasFuasH17 (in short MFH17) was shuffled using the DNaseI-mediated DNA shuffling method described in Ranjan et al. (2012), with few changes. The Muas, Fuas, and H17 fragments were PCR amplified from pUCMuas, pUCFuas, and pUCH17 clones and gel eluted. Around 5 µg of the isolated fragments, namely Muas, Fuas, and H17, were individually treated with 0.5 U of DNaseI enzyme for 10 minutes. The reaction was stopped by adding 0.5 M EDTA and heat-inactivated for 10 minutes. The three reactions, Muas, Fuas, and H17, were mixed and precipitated using 3 M sodium acetate and 99% cold ethanol. The precipitated fragments were then run on 2% agarose gel, and fragments less than 100 bp were eluted. The eluted fragments were then reannealed together using self-priming PCR (primer-less PCR) having the following parameters: 30 seconds at 95°C (denaturation), 30 seconds at 42°C (annealing) and 60 seconds at 72°C (extension) for 25 cycles. Next, the pool of reannealed fragments was used as a PCR reaction template with MFH17-specific primers named Muas F and HRLV R (**Supplementary Data 2** in **Supplementary Data Sheet 1**). The positive PCR products were gel eluted, cloned into pUC119, and sequenced.

### DNA shuffling by oligonucleotide blocks

The oligonucleotide blocks-based DNA shuffling was done following the previously published protocol with small changes (Fujishima et al., 2015). Briefly, twelve pairs (sense and anti-sense strand) of oligos from MFH17 promoter with 40-50 bp in size were synthesized with the addition of AG as 5’ overhang in the sense strand and TC as 5’ overhang in the anti-sense strand (**Supplementary Data 2** in **Supplementary Data Sheet 1**). The sense and anti-sense were then annealed in a thermal cycler by heating at 95°C for 5 minutes and gradually decreasing the temperature to 4°C (1°C/min). The annealed product was then phosphorylated individually using Polynucleotide Kinase (PNK) enzyme and incubated for 30 minutes at 37°C. Parallelly, H17min (HRLV-Flt; 48 bp; -45 to +3; **Supplementary Data 2** in **Supplementary Data Sheet 1**) oligos were also synthesized, which contained Xba1 and HindIII restriction sites in their 5’ and 3’ region, respectively, and annealed, phosphorylated, and cloned into pUC119 vectors leading to the development of pUCH17min. The previously annealed twelve oligos were mixed in a single tube reaction, ligated using T4 DNA ligase enzyme, and kept overnight at 4°C. After incubation, the 5’ and 3’ adapters (5’ containing EcoRI and 3’ XbaI restriction sites; **Supplementary Data 2** in **Supplementary Data Sheet 1**) were added and incubated for another 4-5 hours. Finally, the reaction mixture was cloned into the EcoRI and XbaI sites in the pUCH17min vector. The positive clones were then sequenced and sub-cloned into the plant expression vector pKYLXGUS for further analysis.

The MFH17 promoter contains the H17min sequence (HRLV-Flt; 48 bp; -45 to +3; **Supplementary Data 2** in **Supplementary Data Sheet 1**) as the core promoter region, which is the RNA polymerase binding region. This is why, for developing oligonucleotide blocks shuffled promoters, the H17min sequence was used as the minimal promoter instead of the commonly used 35S minimal promoter (Cai et al., 2020; Yang et al., 2024).

### Transient analysis of developed promoters

All the developed constructs (hybrid, mutant, shuffled) cloned into the pKYLXGUS vector were individually transformed into *Agrobacterium tumefaciens* strain GV3101 using the freeze-thaw method (Weigel and Glazebrook, 2006). For agroinfiltration in *Nicotiana benthamiana*, the positive agrobacterium clones were grown overnight in LB Broth containing Rifampicin and Kanamycin antibiotics. The culture was washed twice and suspended in agro-infiltration buffer (50 mM MES, 2 mM Na_3_PO_4_, 27 mM D-Glucose, and 0.1 mM acetosyringone). The concentration of the agrobacterium culture was kept at OD_600_ 0.1 and infiltrated into the abaxial side of the healthy leaves using a needle-less hypodermic syringe (Mandal et al., 2015). The infiltrated plants were kept in the greenhouse under low-light conditions for 48 hours. The total GUS expression was measured using a fluorometric GUS assay described in Sethi et al. (2022) and Kumari et al. (2024).

For transient analysis in rice seedlings, the positive agrobacterium colonies were grown in LB Broth containing Rifampicin and Kanamycin antibiotics overnight. The culture was then washed and suspended in liquid-infiltration buffer (4g/L Murashige and Skoog (MS) medium, 40 mM KCl, 42 mM MgCl_2_, 200 mM sucrose, 200 mM glucose, 0.01% Silwet and 150 µM acetosyringone) and the culture concentration was adjusted to OD_600_ 1.0. Two weeks old, rice seedlings were submerged into the agrobacterium culture and infiltrated for 10 minutes under 0.9 bar pressure inside the vacuum desiccator. The rice seedlings were washed with distilled water and grown in the greenhouse for 48 hours under low-light conditions. After incubation, GUS expression was measured from the whole seedlings using a fluorometric GUS assay.

For GFP expression analysis, the vectors MuasFuasH17pLXGFP (MFH17), 35SpLXGFP (35S), 2X35SpLXGFP (2X35S), and Ctrl (VC) were transformed into *A. tumefaciens* strain GV3101 and agro-infiltrated into healthy leaves of *N. benthamiana* in small areas. The plants were kept in the greenhouse under low light for 5 days. After incubation, the leaves were detached, viewed under UV light, and photographed using Gel Doc XR+System (Bio-Rad).

### Development and selection of *Nicotiana tabacum* and *Arabidopsis thaliana* transgenic plants

For the development of tobacco transgenic plants, the MuasFuasH17pLXGUS (MFH17), 35SpLXGUS (35S), and Ctrl (VC) plasmids were transformed into *Agrobacterium tumefaciens* strain LBA4404 and the positive colonies were used in developing transgenic plants using leaf disc from *Nicotiana tabacum* cv. Samsun by following the previously published protocol (Horsch et al., 1985). Ten plants were generated from the independent callus and grown in the greenhouse, and each plant’s seeds were harvested and used for seed segregation analysis. Briefly, the seeds were dried and grown in an MS-agar medium containing 200 mg/L Kanamycin for twenty-one days. The Kanamycin-resistant (Kan^R^) and Kanamycin-sensitive (Kan^S^) seedlings were counted and compared with a 3:1 (Kan^R^: Kan^S^) ratio using a *chi*-square test. The lines showing the best phenotype and chi-square value of < 1.5 (p ≤ 0.05) were considered “true transgenic” and grown till the T_2_ generation (Honda et al., 2002; Patro et al., 2015; Chatterjee et al., 2017). Furthermore, the genomic DNA from the selected lines was isolated and used for gene integration PCR, where the promoter (MFH17), gene (*GUS*), Kanamycin-resistant gene (*nptII*), and poly (A) signal (rbcSE9) present in the T-DNA region of pKYLXGUS vector were PCR amplified using their respective primers (**Supplementary Data 2** in **Supplementary Data Sheet 1**).

For the development of Arabidopsis transgenic plants, MuasFuasH17pLXGUS (MFH17), 35SpLXGUS (35S), and Ctrl (VC) plasmids were transformed into *Agrobacterium tumefaciens* strain GV3101 and floral dip method was used to develop *Arabidopsis thaliana* (ecotype Columbia) transgenic plants, following a previously published protocol (Zhang et al., 2006). The infected plant’s seeds were harvested, dried, and germinated in an MS-agar medium containing 50 mg/L Kanamycin. Ten Kanamycin-resistant lines were grown, and their seeds were used for segregation analysis, as described above. A single line with the best phenotype and seed segregation ratio was grown until T_2_ generation, and their gene integration PCR was done as described above.

### Reverse transcription-quantitative PCR

The total RNA from 21-day-old seedlings was isolated using an RNeasy Plant Mini Kit following the manufacturer’s protocol. The isolated RNA was quantitated, and the DNA contamination was purified using the DNaseI enzyme. The purified RNA was then used to make a cDNA pool using the First Strand cDNA Synthesis Kit. Next, around 2 µL (20 ng) of cDNA was then used as a template for RT-PCR reaction containing 10 µL SYBR green master mix, 1 µL forward primer, 1 µL reverse primer and 6 µL nuclease-free water in QuantStudio™5 Real-Time PCR System (Applied Biosystems) having following parameters: 95°C for 10 min, followed by 40 cycles of 95°C for 15s, 60°C for 1 min. The *GUS* transcripts were used as targets, and the *Nt18S* for *Nicotiana tabacum* and *AtActin* for *Arabidopsis thaliana* housekeeping genes were used in normalizing the data [Chanwala et al. (2024); Khan et al. (2018); **Supplementary data 2** in **Supplementary Data Sheet 1**). The data was evaluated using the 2^-ΔΔCT^ method and represented as fold change (Livak and Schmittgen, 2001).

### Histochemical analysis

For detection of GUS activity in different plant tissues, the samples were dipped into an X-Gluc buffer solution containing 50 mM Sodium Phosphate Buffer (pH- 7.0), 0.01% Tween 20, 10 mM EDTA and 0.3% X-Gluc powder and kept at 37°C overnight (12 hours). The sample was then washed, destained in 70% ethanol, and photographed under a Stereomicroscope System (Olympus, SZ61).

### Statistical analysis

All experiments were conducted using three biological replicates, and the average and standard deviation values were utilized for plotting the data. The statistical analysis using the average values was done using the Student’s t-test, where the asterisk ‘*’ sign was used to depict the level of significance (*p ≤ 0.05; **p ≤ 0.01; ***p ≤ 0.001).

## Results

### Analysis of rationally designed hybrid promoters

To develop strong constitutive plant promoters, different fragments from three previously characterized constitutive pararetroviral-based promoters, namely *Mirabilis mosaic virus* (MMV-Flt), *Figwort mosaic virus* (FMV-Flt), and *Horseradish latent virus* (HRLV-Flt) were taken. Two fragments from the HRLV-Flt promoter, namely H17 (250 bp) and H12 (500 bp), were kept as core promoter regions, and three promoter fragments, namely Muas from MMV-Flt, Fuas from FMV-Flt and H17uas from HRLV-Flt were kept as an upstream activation sequence (UAS), functioning as transcriptional enhancers (**Figure 1A**). These three UAS were combined with H17 and H12 core promoter in different combination and ten hybrid promoters were developed, where six were di-hybrids and four were tri-hybrid promoters (**Figure 1B**). The promoter activity analysis of the developed promoter along with H17, H12, 35S, and 2X35S promoters was transiently done in *Nicotiana benthamiana* leaves, and their GUS activity was measured and represented in **Figure 1B**.

The result indicated that all the hybrids were strong and highly active in *N. benthamiana* leaves and showed higher expression than the 35S promoter. The tri-hybrid promoter MuasFuasH17 (in short MFH17), which is a combination of Muas, Fuas, and H17, showed the highest expression, with almost 2 times higher than the 35S promoter and 1.3 times higher than the 2X35S promoter.

The CREs present in the MFH17 promoter were examined using PlantCARE (Lescot et al., 2002) and PLACE (Higo et al., 1999) databases. The sequences were coordinated with numbers, where the transcription start site (TSS) was annotated as +1, and all CREs were numbered accordingly (**Supplementary Data 4** in **Supplementary Data Sheet 1**).

### Analysis of DNaseI-mediated shuffled MFH17 promoter clones

**Figure 2:**
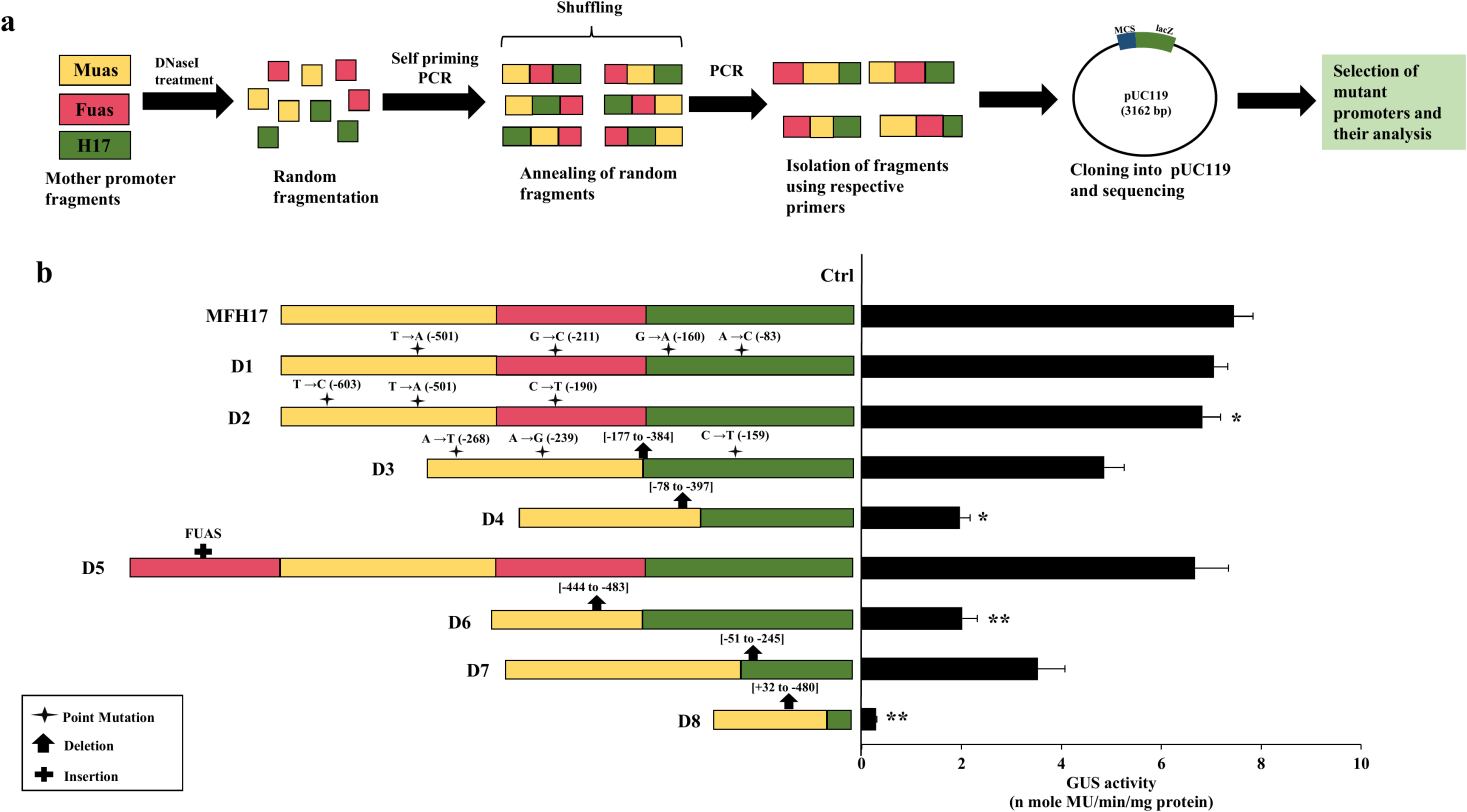
DNaseI-mediated DNA shuffling of MFH17 promoter. **(A)** Assay scheme. The Muas, Fuas, and H17 fragments from the MFH17 promoter were fragmented using the DNaseI enzyme and mixed. The fragments were then annealed using self-priming PCR, and PCR (with primers) was performed from the pool of annealed fragments. The PCR products were then cloned into pUC119 and sequenced. **(B)** Eight mutant clones were generated, viz. D1, D2, D3, D4, D5, D6, D7 and D8, out of which D1 and D2 were point mutant, D3, was both point and deletion mutants, D4, D6, D7 and D8 were deletion mutants and D5 was insertional mutant. All their promoter activity analysis were done in *N. benthamiana* along with MFH17 promoter and Ctrl (VC), and their average GUS activity, with respective SD and statistical significance (*p ≤ 0.05; **p ≤ 0.01), are represented.

It was interesting to observe that even though MuasFuasH17 (MFH17) and FuasMuasH17 promoters shared the same CREs and same promoter domains, and the only variation between them was the position of Muas and Fuas fragments, their promoter activity had significant differences (**Figure 1B**, **Supplementary Data 1** in **Supplementary Data Sheet 1**). Therefore, we attempted to shuffle the promoter fragments of the MFH17 promoter more finely using the directed evolution technique of DNA shuffling to further enhance/upgrade the activity of the MFH17 promoter. The general protocol followed four main steps: 1) fragmentation of Muas, Fuas, and H17 promoters by digestion with DNaseI enzyme, 2) reannealing of these fragments using self-priming PCR (primer-less), 3) selecting of reannealed fragments using PCR (with primer) and 4) cloning, sequencing and screening of unique mutants (**Figure 2A**).

Fifty clones were generated using this technique and sequenced, and we observed that the mutational frequency was very low, as most of the generated clones were unchanged or with only minor point mutations. After screening the sequences, we selected eight mutant clones (D1, D2, D3, D4, D5, D6, D7, and D8) based on the position and type of mutation on the regulatory region of the MFH17 promoter. The D1 and D2 mutant clones had four point mutations and three point mutations, respectively, and caused sequence mutation of MYC (-501; T→A) cis-regulatory elements in D1 and sequence mutation of GATA-motif (-603; T→C) and MYC (-501; T→A) in D2. In the case of the D3 mutant, the whole of the Fuas region was deleted, and a few point mutations were generated, which caused ABRE (-159; C→T) cis-regulatory element mutation. The D4, D6, D7, and D8 were deletion mutants with complete deletion of the Fuas region and partial deletions in the Muas and H17 regions, as depicted in **Figure 2B** (all sequences are provided in **Supplementary Data 1** in **Supplementary Data Sheet 1**). The D5 mutant was an insertion mutation with the addition of a complete Fuas region upstream of the MFH17 promoter (**Figure 2B**).

The promoter activity analysis of all the generated mutant clones was done, and the result revealed that all the mutant clones showed decreased activity compared to the MFH17 promoter. The D1 and D2 mutants, which only had a few point mutations, did not show much difference, whereas all the deletion mutants, such as D3, D4, D6, D7, and D8, showed a significant reduction in the overall activity of MFH17 promoter. Interestingly, the D5 mutant clone with an additional insertion of Fuas enhancer upstream of the MFH17 promoter showed slightly lower expression (**Figure 2B**).

### Analysis of oligo blocks-mediated shuffled MFH17 promoter clones

**Figure 3:**
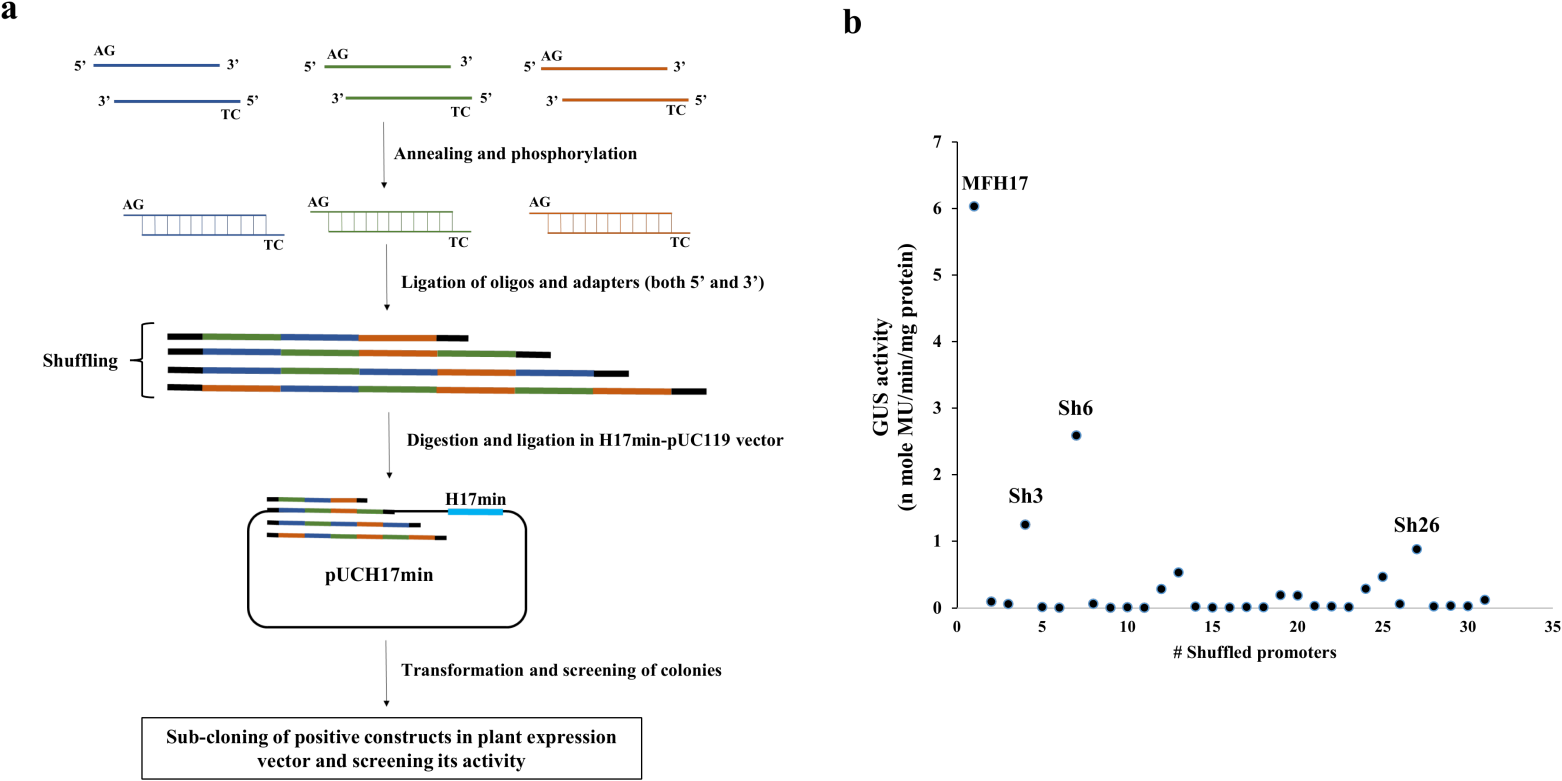
Oligonucleotide-based DNA shuffling. **(A)** Assay scheme. Twelve pairs of 50 bp oligonucleotides from the MFH17 promoter were synthesized, containing AG as a 5’ overhang in the sense strand and TC as a 5’ overhang in the anti-sense strand. These oligos were annealed, phosphorylated, and ligated to one another. The pool of randomly ligated oligos was then cloned into the pUCH17min vector, and positive shuffled promoters were screened by sequencing. Lastly, the positive clones were sub-cloned into the pKYLXGUS vector and its promoter activity analysis was done. **(B)** Thirty shuffled promoters of MFH17 were screened, where only the Sh6 promoter showed moderate expression.

We observed that the DNaseI-mediated shuffling did not yield a “true shuffled” sequence but instead led to either point, deletion, or insertion mutations. Therefore, we attempted another DNA shuffling technique called overhang-based oligonucleotide block shuffling (Fujishima et al., 2015), where 40-50 bp of oligonucleotides sequence from MFH17 promoter were synthesized with sense strand containing AG as 5’ overhang and anti-sense strand containing TC as 5’ overhang as shown in **Figure 3A**. These oligonucleotides were mixed in a single reaction tube and randomly ligated with one another and cloned upstream of H17min promoter (**Figure 3A**). We generated thirty shuffled promoter clones, namely Sh1 to Sh30, and their promoter activity analysis was done.

The result revealed that this technique led to accurate shuffling of DNA sequences, as shown for the sequence of Sh6 (**Supplementary Data 1** in **Supplementary Data Sheet 1**). However, out of the thirty shuffled promoter clones analyzed, most had no expression, and some had weak expressions, such as Sh3 and Sh26. Only the Sh6 shuffled promoter showed moderate expression, around 58% less than the activity of the MFH17 promoter (**Figure 3B**). This might indicate that for proper functioning of a promoter, the regulatory elements must be present in an ideal arrangement. Consequently, the MFH17 promoter is arranged in an optimum arrangement for high-gene expression.

### Transient analysis of MFH17 in rice seedlings and its GFP expression analysis in tobacco leaves

**Figure 4:**
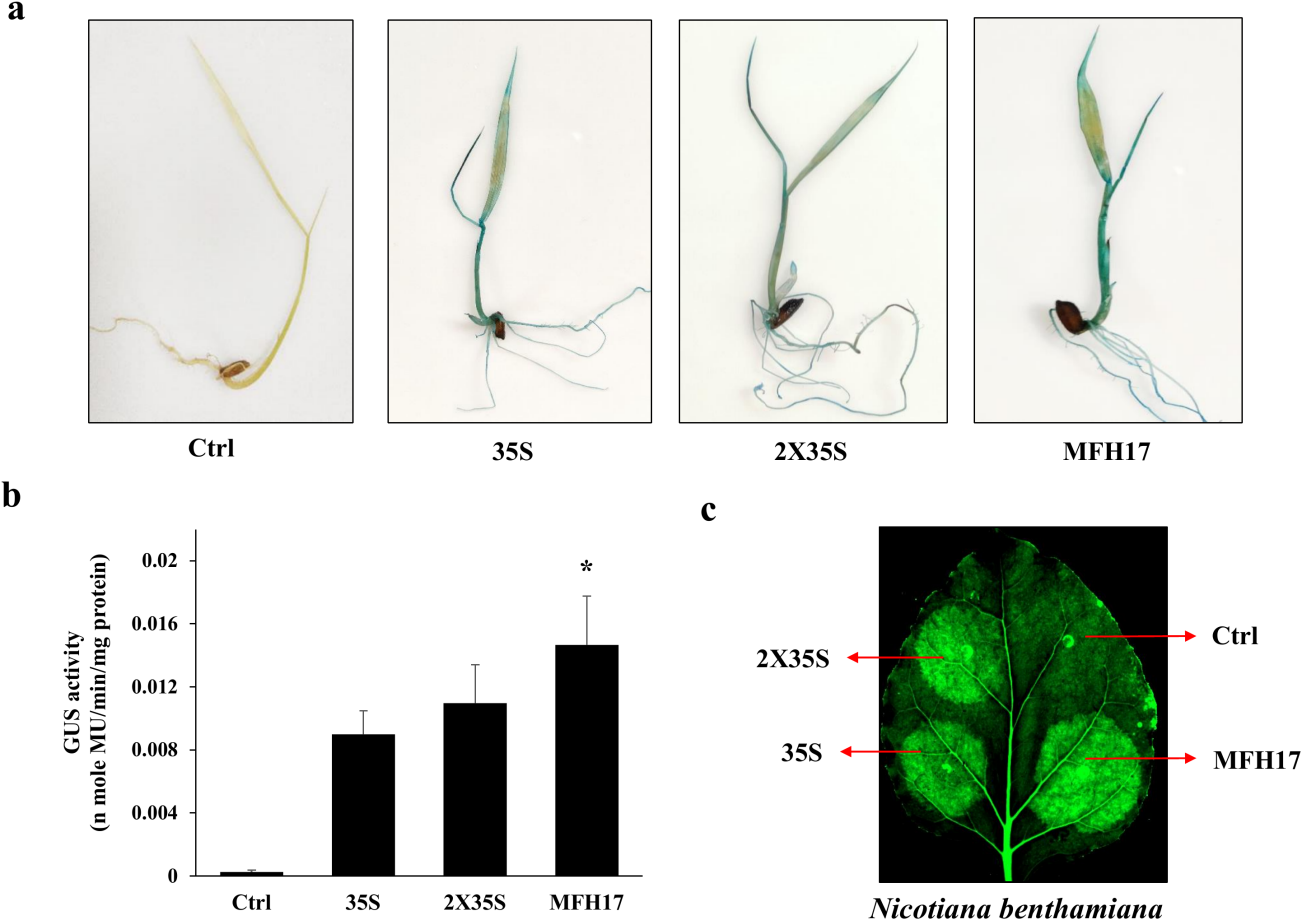
Transient promoter analysis. The promoters MFH17, 35S, and 2X35S with Ctrl (VC) were transiently expressed in rice seedlings, and its **(A)** histochemical staining and **(B)** fluorometric GUS assay were represented, where the asterisk symbol indicates the level of significance. Also, GFP was expressed under MFH17, 35S, and 2X35S promoters with Ctrl (VC) in **(C)** *N. benthamiana* leaves and photographed.

The activity of the MFH17 promoter was further checked in rice seedlings using the *GUS* reporter gene and compared with 35S and 2X35S promoters. The histochemical staining of rice seedlings showed strong blue coloration in all three promoter-infiltrated seedlings, signifying high gene expression (**Figure 4A**). The total GUS activity was quantified from these seedlings, using fluorometric GUS assay and found that similar to promoter activity in *N. benthamiana* leaves, the MFH17 promoter had higher expression in rice seedlings than compared to both 35S and 2X35S promoters (**Figure 4B**).

Next the efficiency of MFH17 in driving GFP was analyzed in *N. benthamiana* leaves. The MFH17, 35S, and 2X35S promoter (driving *GFP* gene) was agro-infiltrated into a small region of *N. benthamiana* leaves and photographed. The images showed intense green fluorescence of GFP expression in the infiltrated regions, signifying that the MFH17 promoter could also efficiently drive the *GFP* reporter gene (**Figure 4C**).

### Effect of as-1 elements on MFH17 promoter activity

**Figure 5:**
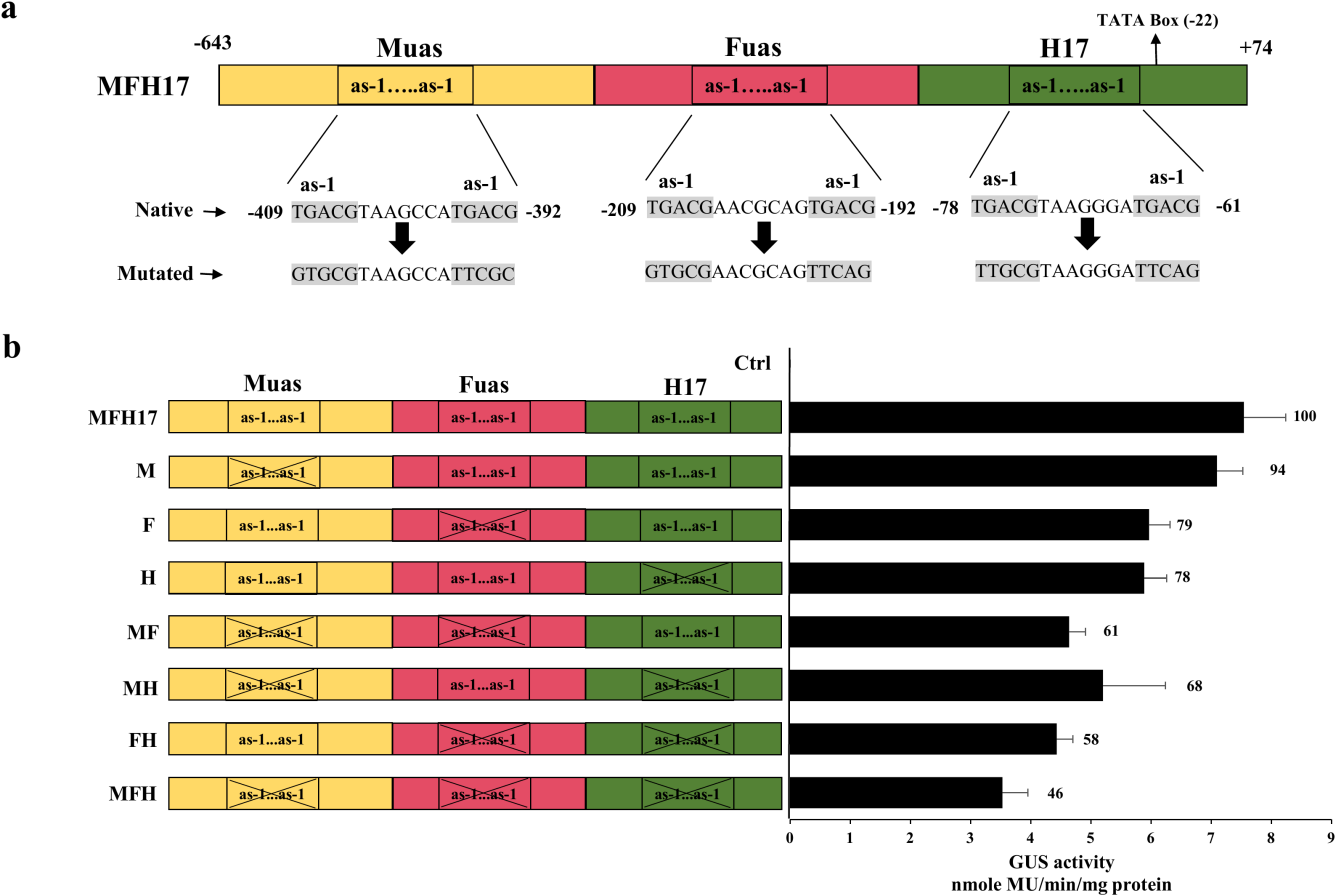
The as-1 elements mutation analysis in MFH17 promoter. **(A)** Schematic representation of positions of as-1 element pairs in MFH17 promoter with its native and mutated sequence. **(B)** The mutated promoter clones with single-pair mutation (M, F, and H), double-pair mutation (MF, MH, and FH), and triple-pair mutation (MFH) were represented, along with MFH17 promoter and Ctrl (VC), and their promoter activity analysis was done. The total average of MFH17 was taken as 100 units, and the averages of other promoters were calculated and represented accordingly.

Previous reports have shown the significance of the as-1 element (TGACG) for the activity of a promoter (Khan et al., 2018; Cai et al., 2020). Our recent work also observed that mutating the as-1 element from the *Horseradish latent virus* sub-genomic transcript promoter led to an almost 75% decrease in its promoter activity (Sherpa et al., 2023). The cis-regulatory element analysis of MFH17 revealed that it contained six as-1 elements (**Supplementary Data 4** in **Supplementary Data Sheet 1**) and were present as pairs in each fragment (Muas, Fuas, and H17) of this promoter (**Figure 5A**). We wanted to check the effect of these as-1 elements pairs in MFH17 promoter activity and therefore did site-directed mutagenesis and generated seven mutant clones, out of which M (as-1 pair mutation in Muas), F (as-1 pair mutation in Fuas), H (as-1 pair mutation in H17) were single pair-mutants, MF (as-1 pair mutations in Muas and Fuas), FH (as-1 pair mutations in Fuas and H17), MH (as-1 pair mutations in Muas and H17) were double pair-mutants and MFH (as-1 pair mutations in Muas, Fuas and H17) was a triple pair-mutant (**Figure 5B**).

The analysis revealed that in the case of the single-pair mutants, the M mutant promoter, which is the most distally present mutation, did not lead to much decrease in the transcriptional activity of the MFH17 promoter, whereas the F and H mutants showed a 21% and 22% decrease, respectively in the activity of MFH17 promoter. The double-pair mutants, MF, MH, and FH, showed more decrease with 39%, 32%, and 42% decline, respectively. The triple-pair mutant MFH promoter showed a 54% decrease in the transcriptional activity of the MFH17 promoter (**Figure 5B**).

### Analysis of MFH17 in transgenic *Nicotiana tabacum* and *Arabidopsis thaliana* plants

To understand the performance of MFH17 *in planta* and study its tissue specificity, transgenic *Nicotiana tabacum* cv. Samsun and *Arabidopsis thaliana* (ecotype Columbia) were developed. Ten independent transgenic lines of tobacco and Arabidopsis containing MFH17 and 35S promoter driving *GUS* reporter gene were developed, and their seed segregation and phenotypic analyses were performed, as described in Material and Methods. For MFH17 promoter analysis in tobacco, line 2 was selected, and for analysis in Arabidopsis, line 4 was selected (**Supplementary Data 5** in **Supplementary Data Sheet 1**). Additionally, genomic DNA was extracted from these two lines, and gene integration PCR was done, which showed distinct bands of MFH17, *GUS*, *nptII*, and rbcSE9 (**Supplementary Data 6** in **Supplementary Data Sheet 1**). These two lines were grown till T_2_ generation, and their analysis was done.

**Figure 6:**
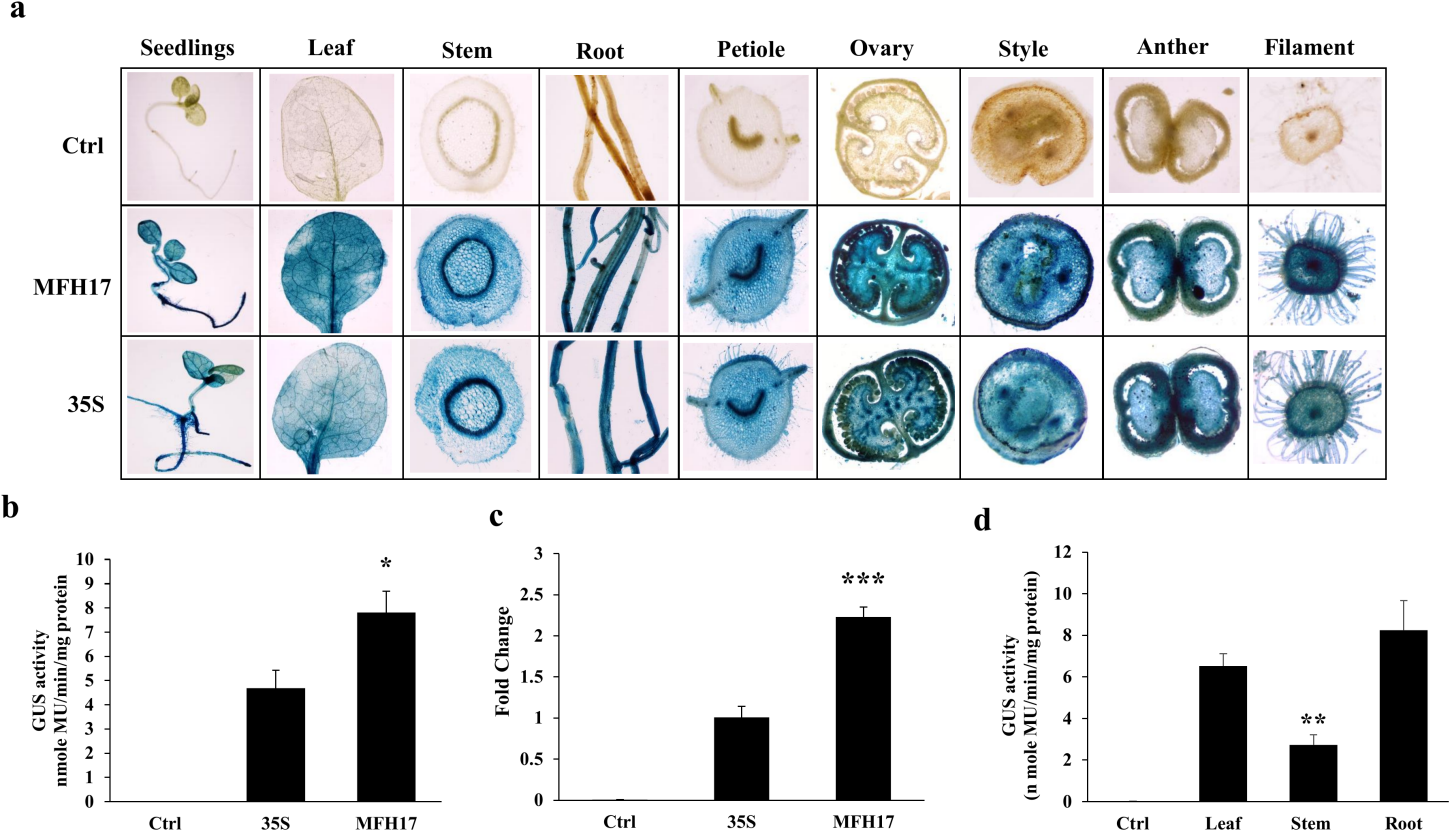
Promoter activity analysis of MFH17, 35S, and Ctrl (VC) in *Nicotiana tabacum* cv. Samsun transgenic plants. **(A)** Histochemical staining of twenty-one-day-old seedlings, forty-five-day-old leaf, stem, root tissues, and ninety-day-old flowering tissues (ovary, style, anther, and filament). **(B)** Fluorometric GUS activity and **(C)** RT-PCR of *GUS* transcript level from twenty-one days old seedlings. **(D)** Fluorometric GUS activity analysis of MFH17 promoter in leaf, stem, and root of forty-five days plants. The asterisk symbol represents the level of significance (*p ≤ 0.05; **p ≤ 0.01; ***p ≤ 0.001).

From tobacco transgenic plants, twenty-one days old seedlings, forty-five days old leaf, stem, root tissues, and ninety days old flowering tissues (ovary, style, anther, and filament) were stained with X-Gluc solution to observe the tissue-specific expression of MFH17 promoter and compared with 35S promoter transgenic plant and control (VC) plant. The result showed that MFH17 and 35S promoters stained deeply in all the tissues, revealing their strong and constitutive nature (**Figure 6A**). We quantitated and compared the activity of MFH17 and 35S promoters by using twenty-one days old seedlings using GUS fluorometric analysis and found that similar to the previous transient analysis, the MFH17 showed stronger activity than 35S, which was approximately 1.7 times higher (**Figure 6B**). Further, the total *uidA (GUS)* transcript level from twenty-one days old seedlings using RT-PCR was measured, and it was found that MFH17 transgenic plants had a higher transcript level than compared to the 35S (**Figure 6C**). We also measured the activity of the MFH17 promoter from the leaf, stem, and root tissue and found it had higher expression in the root than in the leaf or stem (**Figure 6D**).

**Figure 7:**
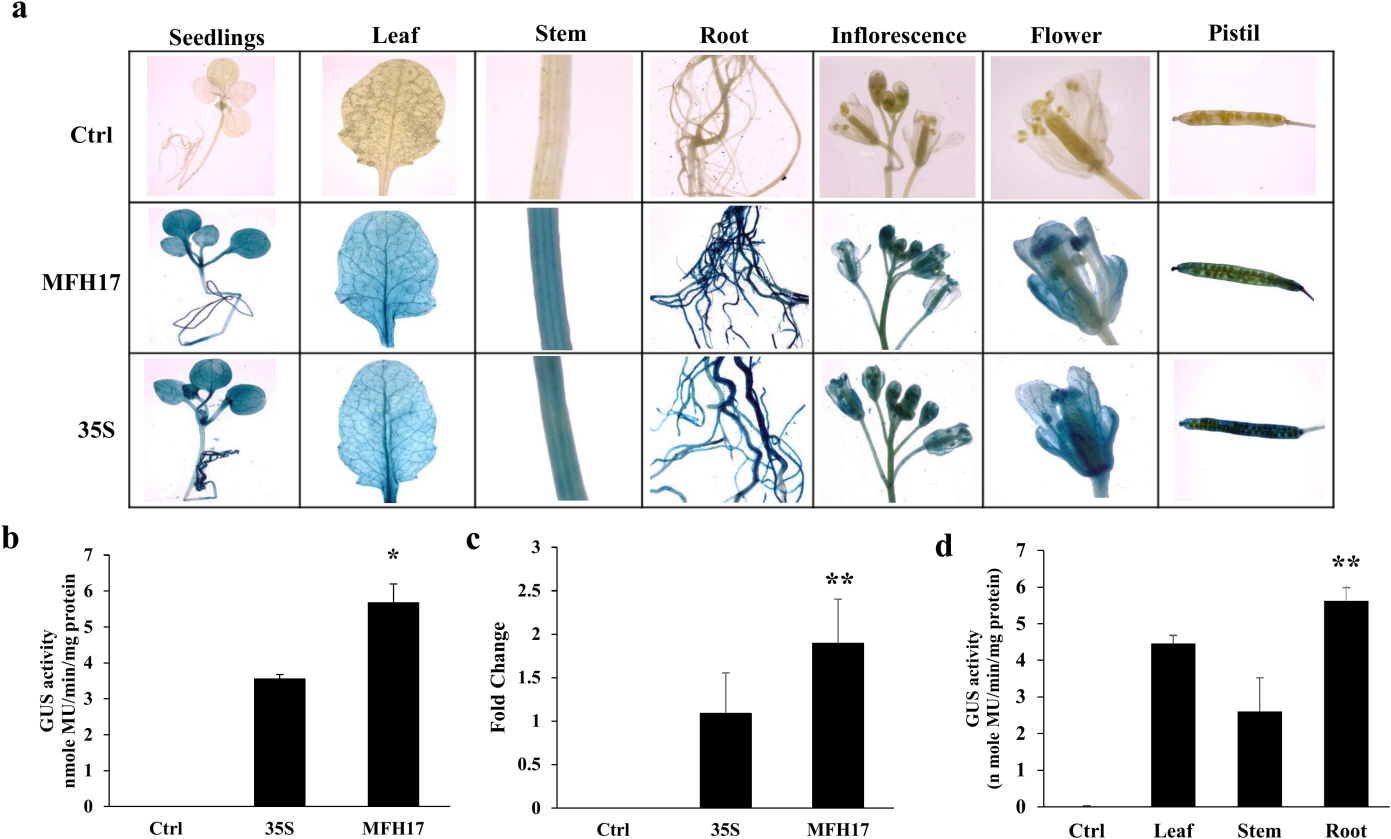
Promoter activity analysis of MFH17, 35S, and Ctrl (VC) in *Arabidopsis thaliana* (ecotype Columbia) transgenic plants. **(A)** Histochemical staining of twenty-one-day-old seedlings, forty-five-day-old leaf, stem, root tissues, and seventy-day-old flowering tissues (inflorescence, flower, and pistil). **(B)** Fluorometric GUS activity and **(C)** RT-PCR of *GUS* transcript level from twenty-one days old seedlings. **(D)** Fluorometric GUS activity analysis of MFH17 promoter in leaf, stem, and root of forty-five days plants. The asterisk symbol represents the level of significance (*p ≤ 0.05; **p ≤ 0.01).

Likewise, in the case of transgenic Arabidopsis plants, twenty-one days old seedlings, forty-five days old leaf, stem, and root tissues, and seventy days old flowering tissues (inflorescence, flower, and pistil) were stained with X-Gluc solution and tissue-specific expression patterns of MFH17 and 35S promoter was observed. The result showed that similar to transgenic tobacco, the MFH17 and 35S promoters stained deeply in all the tissues, showing their strong and constitutive nature (**Figure 7A**). The total promoter activity was quantitated from twenty-one days old seedlings and found that the MFH17 promoter showed 1.6 times higher activity than the 35S promoter (**Figure 7B**). The *GUS* RNA transcript level was also higher in MFH17 Arabidopsis seedlings than compared to the 35S seedlings (**Figure 7C**). Furthermore, the activity of the MFH17 promoter in leaf, stem, and root tissue from forty-five days old transgenic plants was measured, and similar to the expression in tobacco, the promoter showed higher activity in the root than in the leaf and stem (**Figure 7D**).

## Discussion

Over the past decade, biotechnology and synthetic biology have made significant advancements, revolutionizing our understanding and manipulation of biological systems. The emergence of high-throughput biological tools has enabled researchers to conduct larger-scale experiments, facilitating faster data collection and analysis. These biological tools hold tremendous potential for improving healthcare, creating sustainable food sources, and addressing ecological challenges (Patron, 2020; Voigt, 2020). One such tool, plant promoters, is essential for regulating gene expression in plants and plays a critical role in genetic engineering and biotechnology. The selection of an appropriate promoter is essential in any transgenic research to ensure the effective expression of introduced genes. Moreover, the engineering of synthetic promoters enables the customization of gene expression per specific agricultural requirements.

The most widely used method for synthetic promoter development is the rational engineering of synthetic promoter, which depends on the identification or prior knowledge of functional elements (Cai et al., 2020; Deal et al., 2024). The general workflow of rational engineering involves designing promoter constructs by integrating necessary functional domains, assembling them in vectors with suitable reporter modules, screening promoter efficiency in appropriate hosts, and finally analyzing the results (Cai et al., 2020; Einhaus et al., 2021; Yang et al., 2021). Usually, a strong core promoter or a minimal promoter (usually 35S-derived) is hybridized with a desired arrangement of cis-elements or enhancers from different promoter regions. Recently, useful synthetic promoters, such as, SA and JA inducible synthetic promoters, were developed using tailor-made arrangements of responsive cis-regulatory elements (Li et al., 2023); a water-deficit inducible promoter with green tissue specificity was constructed using novel DNA motifs (Yang et al., 2024), and a strong dual-species (dicot and monocot) expressing promoter developed using promoter domains from monocot-specific and dicot-specific promoters (Kumari et al., 2024), have been constructed using this technique. Here, we developed a strong synthetic promoter by rationally combining functional promoter domains from three pararetroviral promoters: *Mirabilis mosaic virus* (MMV)*, Figwort mosaic virus* (FMV), and *Horseradish latent virus* (HRLV). All the hybrid promoters we tested were highly active in *N. benthamiana*, displaying greater activity than the 35S promoter. Among these, the tri-hybrid promoter MuasFuasH17 (MFH17) exhibited the highest expression efficiency, approximately 1.3 times greater than that of the enhanced 35S promoter, known as the 2X35S promoter (**Figure 1B**).

Interestingly, the MuasFuasH17 (MFH17) and FuasMuasH17 tri-hybrid promoters differed in transcriptional activity, even though they shared the same cis-regulatory elements and the same functional domains. The only difference between them was the placement of Muas and Fuas promoter fragments, which might have caused the formation of a new enhanceosome complex through protein-protein interaction in the MFH17 promoter. This observation prompted us to shuffle the sequence of the MFH17 promoter even further using the DNA shuffling method. DNA shuffling is one of the techniques of directed evolution where high genetic diversification is done on DNA sequences through a series of mutations, and the library of mutated promoters is screened for important functional traits (Wang et al., 2021). Unlike the rational engineering method, where the outcome of a system can be predicted, directed evolution results in the generation of random mutations in DNA sequence, which cannot be predicted and, therefore, can lead to a library of promoters with unique features (Rao et al., 2021). Directed evolution is a powerful technique and has been successfully used in many protein engineering projects, where proteins with improved catalytic efficiency, improved resistance to certain inhibitors, optimized solubility of enzymes, and increased fluorescence have been developed (Sawano and Miyawaki, 2000; Fan et al., 2010; Rao et al., 2021; McLure et al., 2022). However, this technique has not been widely used in promoter engineering projects. Here, we first attempted the DNaseI-mediated DNA shuffling technique; however, most of the clones we got were identical to the mother promoter fragments, and only eight clones were mutated, with either point, deletion or insertional mutations (4% mutation frequency). Such limitation of this technique for generating mutant sequences has also been previously reported (Stemmer, 1994; Ranjan et al., 2012; Wang et al., 2021). A few point mutations (D1 and D2) did not change the MFH17 promoter activity a lot, but all deletion mutants (D3, D4, D6, D7, and D8) significantly impacted the promoter activity. The insertional mutant D5, which had an additional insertion of Fuas enhancer upstream of the MFH17 promoter, also did not significantly impact MFH17 promoter activity, which might suggest the limit to which the addition of upstream activation sequences (UAS) affects the promoter activity (**Figure 2B**). We attempted another DNA shuffling technique called overhang-based DNA block shuffling, and this technique successfully generated “true shuffled” sequences of the MFH17 promoter, but almost all the shuffled promoters showed weak to zero expression efficiency, with only the Sh6 promoter showing moderate activity (**Figure 3B**). These results suggest that the rational design method may be superior to directed evolution in developing synthetic promoters.

Further functional characterization of the MFH17 promoter revealed that it was highly active in rice seedlings and showed higher activity than 35S and 2X35S promoters (**Figure 4A**). The MFH17 promoter was also effective in producing high levels of GFP in tobacco leaves (**Figure 4C**), signifying the efficiency of the MFH17 promoter in expressing genes in both dicot and monocot plant cells. We checked the presence of cis-regulatory elements in the MFH17 promoter and found a high number of as-1 elements. The as-1 elements have also been found to be present in many pararetroviral promoters, such as MMV, FMV, DaMV, HRLV, CaMV, and PClSV (Maiti et al., 1997; Dey and Maiti, 1999; Krawczyk et al., 2002; Sahoo et al., 2015; Khan et al., 2018; Sherpa et al., 2023). These elements have been shown to interact with the TGA family of transcription factors, which belongs to the basic region leucine zipper (bZIP) family of transcription factors and have a broad functional role in stress response, detoxification, developmental processes, etc (Tomaž et al., 2022). Some bZIP transcription factors are thought to play a role in relaxing chromatin compaction (Hörberg and Reymer, 2020). This could help explain the high expression efficiency of the MFH17 promoter. When these bZIP transcription factors bind to the as-1 elements, the promoter region becomes less compact, allowing for the assembly of more transcription factors. This hypothesis is further supported by mutational analyses of the as-1 elements within the MFH17 promoter. As the number of mutations in these as-1 elements increases, the activity of the promoter significantly declines. This experiment also provides valuable insight into the positioning of as-1 elements in the development of synthetic promoters. Our observations indicate that mutating as-1 elements located close to the core promoter region (mutant F and mutant H) had a greater impact on MFH17 activity compared to mutating the more distant as-1 elements (mutant M) (**Figure 5B**). Therefore, for the development of robust synthetic promoters, it may be optimal to place as-1 elements within 50 to 250 base pairs upstream of the transcription start site.

The 35S promoter is extensively utilized in plant biology and has been comprehensively characterized across various plant species. This viral promoter is known for its high transcriptional activity and ability to provide constitutive expression in most tissues, with few exceptions (Benfey and Chua, 1990; Sunilkumar et al., 2002; Auriac and Timmers, 2007). Consequently, it has been suggested to be used as a standard reference promoter for plant synthetic biology experiments (Amack and Antunes, 2020; Amack et al., 2023). Here, we developed and studied the activity of MFH17 and 35S promoter in both *Nicotiana tabacum* and *Arabidopsis thaliana* transgenic plants and compared their expression profile. The MFH17 promoter, similar to the 35S promoter, was highly constitutive as it showed the development of intense color in all the parts (both vegetative and reproductive) stained (**Figures 6A**, **7A**). The overall transcriptional activity of the MFH17 promoter was found to be significantly higher than that of the 35S promoter in both *Nicotiana tabacum* and *Arabidopsis thaliana* transgenic plants (**Figures 6B**, **7B**). Our findings indicate that the MFH17 promoter is strong, highly constitutive, and can be effectively utilized in both transient and transgenic systems to achieve high levels of gene expression.

Strong constitutive promoters enable continuous and stable expression of transgenes in different tissues and developmental stages, making them valuable for producing proteins consistently throughout a plant’s lifecycle. Such promoters are the most widely used type of plant promoters and have been essential in different biotechnological applications for generating recombinant proteins, enzymes, and other important biomolecules in plants. Our previous works where a strong constitutive promoter, M24, was used to express the rat Par-4-SAC protein in transgenic tobacco plants showed that the purified protein significantly delayed tumor growth in a rat prostate cancer model, highlighting the significance of strong promoters in the molecular farming of therapeutic proteins (Sarkar et al., 2015). Strong constitutive promoters have also been used to enhance plant resistance by expressing recombinant anti-microbial proteins. The synthetic promoter MUASCsV8CP, which has activity similar to the 2X35S promoter, expressed Killer Protein 4 (KP4) in transgenic tobacco plants, leading to increased tolerance to the fungi *Alternaria alternata* and *Phoma exigua* (Deb et al., 2018). Additionally, these types of promoters have also been employed to develop stress-resilient plants by overexpressing stress-related proteins. A recent example includes the overexpression of the pearl millet transcription factor, PgWRKY44, in *Arabidopsis thaliana* using the 35S promoter, which led to transgenic plants showing improved tolerance to various abiotic stresses, such as drought and salt (Chanwala et al., 2024).

However, there are also various cases where the overexpression of certain proteins has led to significant negative changes in the phenotype of the host plants. For example, in transgenic plants where DREB1A was overexpressed under the control of the 35S promoter, the plants exhibited severely retarded growth (Kasuga et al., 1999). Therefore, though the constitutive promoters offer several benefits, such as stable expression and ease of use, their inherent inability to regulate gene expression requires careful consideration in different genetic engineering projects. Before utilizing strong constitutive promoters for gene expression studies, it is essential to thoroughly assess the biochemical and biophysical properties of the target protein (Egelkrout et al., 2012). This evaluation is crucial to determine if overexpression could disrupt cellular metabolic pathways and potentially jeopardize the organism’s homeostasis.

## Conclusion

In this work, we rationally designed and developed a plant synthetic promoter, MFH17, by combining three promoter fragments from pararetroviruses *Mirabilis mosaic virus* (MMV), *Figwort mosaic virus* (FMV) and *Horseradish latent virus* (HRLV). We observed that rational design is more powerful for developing synthetic promoters than DNA shuffling (directed evolution) techniques. We also studied the effect of as-1 elements of the MFH17 promoter using site-directed mutagenesis and further validated the efficacy of the MFH17 promoter in rice and tobacco plants and its tissue specificity in transgenic *Nicotiana tabacum* and *Arabidopsis thaliana*. Finally, we concluded that the MFH17 promoter is strong and efficiently able to drive genes in both dicot and monocot plants; furthermore, this promoter is also highly constitutive in transgenic plants and could be an important addition to the constitutive plant promoter library.

## Data Availability

The data generated in the study are included in the article/**Supplementary Material**. Further inquiries can be directed to the corresponding author.
