# Supplementary material for: Development of robust constitutive synthetic promoter using genetic resources of plant pararetroviruses
Source: Front Plant Sci. 2025 Jan 22;15:1515921. doi: 10.3389/fpls.2024.1515921 (PMC11794816; doi:10.3389/fpls.2024.1515921)
Supplement: Supplementary file 1 [file DataSheet1.pdf]

Supplementary Materials

Development of robust constitutive synthetic promoter using genetic resources of plant pararetrovirus

Tsheten Sherpa[a,b], Nrisingha Dey[a#]

Affiliation:

[a] **Division of Plant Biotechnology, Institute of Life Sciences,** NALCO Square, Chandrasekharpur, Bhubaneswar, Odisha 751023

[b] **Regional Centre for Biotechnology,** National Capital Region Biotech Science Cluster, Faridabad, Haryana (NCR Delhi) 121001

Email: TS: tshetensherpa70@gmail.com, **ND**: nrisinghad@gmail.com

# Corresponding Author

Dr. Nrisingha Dey,

Phone: (+91)-674-2300728

Mobile: (+91)-9937163453

Supplementary Data 1:

Sequences of mother and hybrid promoters, where TATA Box have been labeled in red and TSS as bold yellow highlighted.

>MuasFuasH17cp- MFH17 (716 bp; 5'→3')

TTCGTCCACAGACATCAACATCTTATCGTCCTTTGAAGATAAGATAATAATGTTGAAGAT
AAGAGTGGGAGCCACCACTAAAACATTGCTTTGTCAAAAGCTAAAAAAGATGATGCCCG
ACAGCCACTTGTGTGAAGCATGTGAAGCCGGTCCCTCCACTAAGAAAATTAGTGAAGCA
TCTTCCAGTGGTCCCTCCACTCACAGCTCAATCAGTGAGCAACAGGACGAAGGAAATGA
CGTAAGCCATGACGTCTAATCCCCCGACAGCTGGCTTGTGGGGACCAGACAAAAAAGGA
ATGGTGCAGAATTGTTAGGCGCACCTACCAAAAGCATCTTTGCCTTTATTGCAAAGATAA
AGCAGATTCCTCTAGTACAAGTGGGGAACAAAATAACGTGGAAAAGAGCTGTCCTGACA
GCCCACTCACTAATGCGTATGACGAACGCAGTGACGACCACAAAACCCGACCAATGGAG
GGCCCCAGACACGTGAAGACGCGTCTGCCGACAGTGGGTCTCGGACAACAGACACCACG
CACTCAGCAAGTGGATGAAATAATTCATCTGCTGACGTAAGGGATGACGATCAATCCCA
CTATCCCAAGACCCTTCACTTCTATATAAGTGAAGTTGCTTCATTTGGAGA**A**GGCATCTC
GAAATCTCAACACAACTCGAGCTCTCCCTTCTCTCTTCTTTATCTCTCTAAATGTGTGAGT
AGA

>H12 (500 bp; 5'→3')
TGGTAGACTATGAAACACTAGTCTACTCAAAGAACTTGAAGAAGACGACTCAGGAAGAC
AGGAGCGTCATCAACAAGTTTCAGCAAAAGCTGATTAGTGGAAAAATCCTTGGATTCCA
CTCTCCAGCAATCTGCCAGCACATAAAGGTGACAGCAGAAAAAGAAGATTGTGACTACC
ACTGCAATCAGTGCGAATCTTCAAAAGGAAAGGCTATCGTTTGCGATAAGCCTGCCGAC
AGTGGTCCAGCCGACAATGGAGGGCCCCAGACACGTGAAGACGCGTCTGCCGACAGTGG
GTCTCGGACAACAGACACCACGCACTCAGCAAGTGGATGAAATAATTCATCTGCTGACG
TAAGGGATGACGATCAATCCCACTATCCCAAGACCCTTCACTTCTATATAAGTGAAGTTG
CTTCATTTGGAGA**A**GGCATCTCGAAATCTCAACACAACTCGAGCTCTCCCTTCTCTCTTCT
TTATCTCTCTAAATGTGTGAGTAGA

>H17 (250 bp; 5'→3')

CAATGGAGGGCCCCAGACACGTGAAGACGCGTCTGCCGACAGTGGGTCTCGGACAACAG
ACACCACGCACTCAGCAAGTGGATGAAATAATTCATCTGCTGACGTAAGGGATGACGAT
CAATCCCACTATCCCAAGACCCTTCACTTCTATATAAGTGAAGTTGCTTCATTTGGAGA**A**
GGCATCTCGAAATCTCAACACAACTCGAGCTCTCCCTTCTCTCTTCTTTATCTCTCTAAAT
GTGTGAGTAGA

>H17uas (148 bp; 5'→3')
CAATGGAGGGCCCCAGACACGTGAAGACGCGTCTGCCGACAGTGGGTCTCGGACAACAG
ACACCACGCACTCAGCAAGTGGATGAAATAATTCATCTGCTGACGTAAGGGATGACGAT
CAATCCCACTATCCCAAGACCCTTCACTTC

>**Muas** (259 bp; 5'→3')

TTCGTCCACAGACATCAACATCTTATCGTCCTTTGAAGATAAGATAATAATGTTGAAGAT
AAGAGTGGGAGCCACCACTAAAACATTGCTTTGTCAAAAGCTAAAAAAGATGATGCCCG

ACAGCCACTTGTGTGAAGCATGTGAAGCCGGTCCCTCCACTAAGAAAATTAGTGAAGCA
TCTTCCAGTGGTCCCTCCACTCACAGCTCAATCAGTGAGCAACAGGACGAAGGAAATGA
CGTAAGCCATGACGTCTAATCC

>Fuas (195 bp; 5’→3’)

AGCTGGCTTGTGGGGACCAGACAAAAAAGGAATGGTGCAGAATTGTTAGGCGCACCTAC
CAAAAGCATCTTTGCCTTTATTGCAAAGATAAAGCAGATTCCTCTAGTACAAGTGGGGAA
CAAAATAACGTGGAAAAGAGCTGTCCTGACAGCCCACTCACTAATGCGTATGACGAACG
CAGTGACGACCACAAAA

>H17uasH17 (404 bp; 5’→3’)

CAATGGAGGGCCCCAGACACGTGAAGACGCGTCTGCCGACAGTGGGTCTCGGACAACAG
ACACCACGCACTCAGCAAGTGGATGAAATAATTCATCTGCTGACGTAAGGGATGACGAT
CAATCCCACTATCCCAAGACCCTTCACTTCCCCGACCAATGGAGGGCCCCAGACACGTGA
AGACGCGTCTGCCGACAGTGGGTCTCGGACAACAGACACCACGCACTCAGCAAGTGGAT
GAAATAATTCATCTGCTGACGTAAGGGATGACGATCAATCCCACTATCCCAAGACCCTTC
ACTTCTATATAAGTGAAGTTGCTTCATTTGGAGA**A**GGCATCTCGAAATCTCAACACAACT
CGAGCTCTCCCTTCTCTCTTCTTTATCTCTCTAAATGTGTGAGTAGA

>FuasH17cp (451 bp; 5’→3’)

AGCTGGCTTGTGGGGACCAGACAAAAAAGGAATGGTGCAGAATTGTTAGGCGCACCTAC
CAAAAGCATCTTTGCCTTTATTGCAAAGATAAAGCAGATTCCTCTAGTACAAGTGGGGAA
CAAAATAACGTGGAAAAGAGCTGTCCTGACAGCCCACTCACTAATGCGTATGACGAACG
CAGTGACGACCACAAAACCCGACCAATGGAGGGCCCCAGACACGTGAAGACGCGTCTGC
CGACAGTGGGTCTCGGACAACAGACACCACGCACTCAGCAAGTGGATGAAATAATTCAT
CTGCTGACGTAAGGGATGACGATCAATCCCACTATCCCAAGACCCTTCACTTCTATATAA
GTGAAGTTGCTTCATTTGGAG**A**AGGCATCTCGAAATCTCAACACAACTCGAGCTCTCCCT
TCTCTCTTCTTTATCTCTCTAAATGTGTGAGTAGA

>MuasH17cp (515 bp; 5’→3’)

TTCGTCCACAGACATCAACATCTTATCGTCCTTTGAAGATAAGATAATAATGTTGAAGAT
AAGAGTGGGAGCCACCACTAAAACATTGCTTTGTCAAAAGCTAAAAAAGATGATGCCCG
ACAGCCACTTGTGTGAAGCATGTGAAGCCGGTCCCTCCACTAAGAAAATTAGTGAAGCA
TCTTCCAGTGGTCCCTCCACTCACAGCTCAATCAGTGAGCAACAGGACGAAGGAAATGA
CGTAAGCCATGACGTCTAATCCCCCGACCAATGGAGGGCCCCAGACACGTGAAGACGCG
TCTGCCGACAGTGGGTCTCGGACAACAGACACCACGCACTCAGCAAGTGGATGAAATAA
TTCATCTGCTGACGTAAGGGATGACGATCAATCCCACTATCCCAAGACCCTTCACTTCTA
TATAAGTGAAGTTGCTTCATTTGGAGA**A**GGCATCTCGAAATCTCAACACAACTCGAGCTC
TCCCTTCTCTCTTCTTTATCTCTCTAAATGTGTGAGTAGA

>FuasMuasH17cp (716 bp; 5’→3’)
AGCTGGCTTGTGGGGACCAGACAAAAAAGGAATGGTGCAGAATTGTTAGGCGCACCTAC
CAAAAGCATCTTTGCCTTTATTGCAAAGATAAAGCAGATTCCTCTAGTACAAGTGGGGAA
CAAAATAACGTGGAAAAGAGCTGTCCTGACAGCCCACTCACTAATGCGTATGACGAACG
CAGTGACGACCACAAAACCCGACTTCGTCCACAGACATCAACATCTTATCGTCCTTTGAA
GATAAGATAATAATGTTGAAGATAAGAGTGGGAGCCACCACTAAAACATTGCTTTGTCA
AAAGCTAAAAAAGATGATGCCCGACAGCCACTTGTGTGAAGCATGTGAAGCCGGTCCCT

CCACTAAGAAAATTAGTGAAGCATCTTCCAGTGGTCCCTCCACTCACAGCTCAATCAGTG
AGCAACAGGACGAAGGAAATGACGTAAGCCCCCGACATGACGTCTAATCCCAATGGAGG
GCCCCAGACACGTGAAGACGCGTCTGCCGACAGTGGGTCTCGGACAACAGACACCACGC
ACTCAGCAAGTGGATGAAATAATTCATCTGCTGACGTAAGGGATGACGATCAATCCCAC
TATCCCAAGACCCTTCACTTCTATATAAGTGAAGTTGCTTCATTTGGAGA**A**GGCATCTCG
AAATCTCAACACAACTCGAGCTCTCCCTTCTCTCTTCTTTATCTCTCTAAATGTGTGAGTA
GA

> **FuasH12cp** (701 bp; 5’→3’)

AGCTGGCTTGTGGGGACCAGACAAAAAAGGAATGGTGCAGAATTGTTAGGCGCACCTAC
CAAAAGCATCTTTGCCTTTATTGCAAAGATAAAGCAGATTCCTCTAGTACAAGTGGGGAA
CAAAATAACGTGGAAAAGAGCTGTCCTGACAGCCCACTCACTAATGCGTATGACGAACG
CAGTGACGACCACAAAACCCGACTGGTAGACTATGAAACACTAGTCTACTCAAAGAACT
TGAAGAAGACGACTCAGGAAGACAGGAGCGTCATCAACAAGTTTCAGCAAAAGCTGATT
AGTGGAAAAATCCTTGGATTCCACTCTCCAGCAATCTGCCAGCACATAAAGGTGACAGC
AGAAAAAGAAGATTGTGACTACCACTGCAATCAGTGCGAATCTTCAAAAGGAAAGGCTA
TCGTTTGCGATAAGCCTGCCGACAGTGGTCCAGCCGACAATGGAGGGCCCCAGACACGT
GAAGACGCGTCTGCCGACAGTGGGTCTCGGACAACAGACACCACGCACTCAGCAAGTGG
ATGAAATAATTCATCTGCTGACGTAAGGGATGACGATCAATCCCACTATCCCAAGACCCT
TCACTTCTATATAAGTGAAGTTGCTTCATTTGGAGA**A**GGCATCTCGAAATCTCAACACAA
CTCGAGCTCTCCCTTCTCTCTTCTTTATCTCTCTAAATGTGTGAGTAGA

>**MuasH12cp** (765 bp; 5’→3’)

TTCGTCCACAGACATCAACATCTTATCGTCCTTTGAAGATAAGATAATAATGTTGAAGAT
AAGAGTGGGAGCCACCACTAAAACATTGCTTTGTCAAAAGCTAAAAAAGATGATGCCCG
ACAGCCACTTGTGTGAAGCATGTGAAGCCGGTCCCTCCACTAAGAAAATTAGTGAAGCA
TCTTCCAGTGGTCCCTCCACTCACAGCTCAATCAGTGAGCAACAGGACGAAGGAAATGA
CGTAAGCCCCCGACATGACGTCTAATCCTGGTAGACTATGAAACACTAGTCTACTCAAAG
AACTTGAAGAAGACGACTCAGGAAGACAGGAGCGTCATCAACAAGTTTCAGCAAAAGCT
GATTAGTGGAAAAATCCTTGGATTCCACTCTCCAGCAATCTGCCAGCACATAAAGGTGAC
AGCAGAAAAAGAAGATTGTGACTACCACTGCAATCAGTGCGAATCTTCAAAAGGAAAGG
CTATCGTTTGCGATAAGCCTGCCGACAGTGGTCCAGCCGACAATGGAGGGCCCCAGACA
CGTGAAGACGCGTCTGCCGACAGTGGGTCTCGGACAACAGACACCACGCACTCAGCAAG
TGGATGAAATAATTCATCTGCTGACGTAAGGGATGACGATCAATCCCACTATCCCAAGAC
CCTTCACTTCTATATAAGTGAAGTTGCTTCATTTGGAGA**A**GGCATCTCGAAATCTCAACA
CAACTCGAGCTCTCCCTTCTCTCTTCTTTATCTCTCTAAATGTGTGAGTAGA

>**FuasMuasH12cp** (966 bp; 5’→3’)
AGCTGGCTTGTGGGGACCAGACAAAAAAGGAATGGTGCAGAATTGTTAGGCGCACCTAC
CAAAAGCATCTTTGCCTTTATTGCAAAGATAAAGCAGATTCCTCTAGTACAAGTGGGGAA
CAAAATAACGTGGAAAAGAGCTGTCCTGACAGCCCACTCACTAATGCGTATGACGAACG
CAGTGACGACCACAAAACCCGACTTCGTCCACAGACATCAACATCTTATCGTCCTTTGAA
GATAAGATAATAATGTTGAAGATAAGAGTGGGAGCCACCACTAAAACATTGCTTTGTCA
AAAGCTAAAAAAGATGATGCCCGACAGCCACTTGTGTGAAGCATGTGAAGCCGGTCCCT
CCACTAAGAAAATTAGTGAAGCATCTTCCAGTGGTCCCTCCACTCACAGCTCAATCAGTG

AGCAACAGGACGAAGGAAATGACGTAAGCCCCCGACATGACGTCTAATCCTGGTAGACT
ATGAAACACTAGTCTACTCAAAGAACTTGAAGAAGACGACTCAGGAAGACAGGAGCGTC
ATCAACAAGTTTCAGCAAAAGCTGATTAGTGGAAAAATCCTTGGATTCCACTCTCCAGCA
ATCTGCCAGCACATAAAGGTGACAGCAGAAAAAGAAGATTGTGACTACCACTGCAATCA
GTGCGAATCTTCAAAAGGAAAGGCTATCGTTTGCGATAAGCCTGCCGACAGTGGTCCAG
CCGACAATGGAGGGCCCCAGACACGTGAAGACGCGTCTGCCGACAGTGGGTCTCGGACA
ACAGACACCACGCACTCAGCAAGTGGATGAAATAATTCATCTGCTGACGTAAGGGATGA
CGATCAATCCCACTATCCCAAGACCCTTCACTTCTATATAAGTGAAGTTGCTTCATTTGGA
GA**A**GGCATCTCGAAATCTCAACACAACTCGAGCTCTCCCTTCTCTCTTCTTTATCTCTCTA
AATGTGTGAGTAGA

>**MuasFuasH12cp** (966 bp; 5’→3’)
TTCGTCCACAGACATCAACATCTTATCGTCCTTTGAAGATAAGATAATAATGTTGAAGAT
AAGAGTGGGAGCCACCACTAAAACATTGCTTTGTCAAAAGCTAAAAAAGATGATGCCCG
ACAGCCACTTGTGTGAAGCATGTGAAGCCGGTCCCTCCACTAAGAAAATTAGTGAAGCA
TCTTCCAGTGGTCCCTCCACTCACAGCTCAATCAGTGAGCAACAGGACGAAGGAAATGA
CGTAAGCCCCCGACATGACGTCTAATCCAGCTGGCTTGTGGGGACCAGACAAAAAAGGA
ATGGTGCAGAATTGTTAGGCGCACCTACCAAAAGCATCTTTGCCTTTATTGCAAAGATAA
AGCAGATTCCTCTAGTACAAGTGGGGAACAAAATAACGTGGAAAAGAGCTGTCCTGACA
GCCCACTCACTAATGCGTATGACGAACGCAGTGACGACCACAAAACCCGACTGGTAGAC
TATGAAACACTAGTCTACTCAAAGAACTTGAAGAAGACGACTCAGGAAGACAGGAGCGT
CATCAACAAGTTTCAGCAAAAGCTGATTAGTGGAAAAATCCTTGGATTCCACTCTCCAGC
AATCTGCCAGCACATAAAGGTGACAGCAGAAAAAGAAGATTGTGACTACCACTGCAATC
AGTGCGAATCTTCAAAAGGAAAGGCTATCGTTTGCGATAAGCCTGCCGACAGTGGTCCA
GCCGACAATGGAGGGCCCCAGACACGTGAAGACGCGTCTGCCGACAGTGGGTCTCGGAC
AACAGACACCACGCACTCAGCAAGTGGATGAAATAATTCATCTGCTGACGTAAGGGATG
ACGATCAATCCCACTATCCCAAGACCCTTCACTTCTATATAAGTGAAGTTGCTTCATTTGG
AGA**A**GGCATCTCGAAATCTCAACACAACTCGAGCTCTCCCTTCTCTCTTCTTTATCTCTCT
AAATGTGTGAGTAGA

>**H17uasH12** (654 bp; 5’→3’)
CAATGGAGGGCCCCAGACACGTGAAGACGCGTCTGCCGACAGTGGGTCTCGGACAACAG
ACACCACGCACTCAGCAAGTGGATGAAATAATTCATCTGCTGACGTAAGGGATGACGAT
CAATCCCACTATCCCAAGACCCTTCACTTCCCCGACTGGTAGACTATGAAACACTAGTCT
ACTCAAAGAACTTGAAGAAGACGACTCAGGAAGACAGGAGCGTCATCAACAAGTTTCAG
CAAAAGCTGATTAGTGGAAAAATCCTTGGATTCCACTCTCCAGCAATCTGCCAGCACATA
AAGGTGACAGCAGAAAAAGAAGATTGTGACTACCACTGCAATCAGTGCGAATCTTCAAA
AGGAAAGGCTATCGTTTGCGATAAGCCTGCCGACAGTGGTCCAGCCGACAATGGAGGGC
CCCAGACACGTGAAGACGCGTCTGCCGACAGTGGGTCTCGGACAACAGACACCACGCAC
TCAGCAAGTGGATGAAATAATTCATCTGCTGACGTAAGGGATGACGATCAATCCCACTAT
CCCAAGACCCTTCACTTCTATATAAGTGAAGTTGCTTCATTTGGAGA**A**GGCATCTCGAAA
TCTCAACACAACTCGAGCTCTCCCTTCTCTCTTCTTTATCTCTCTAAATGTGTGAGTAGA

Sequences of DNaseI mediated shuffled clones, where point mutation is highlighted in blue, TATA Box has been labeled in red, and TSS is bold yellow highlighted.

>D1 (716 bp; 5'→3')

TTCGTCCACAGACATCAACATCTTATCGTCCTTTGAAGATAAGATAATAATGTTGAAGAT
AAGAGTGGGAGCCACCACTAAAACATTGCTTTGTCAAAAGCTAAAAAAGATGATGCCCG
ACAGCCACTTGTGTGAAGCATGAGAAGCCGGTCCCTCCACTAAGAAAATTAGTGAAGCA
TCTTCCAGTGGTCCCTCCACTCACAGCTCAATCAGTGAGCAACAGGACGAAGGAAATGA
CGTAAGCCATGACGTCTAATCCAGCTGGCTTGTGGGGACCAGACAAAAAAGGAATGGTG
CAGAATTGTTAGGCGCACCTACCAAAAGCATCTTTGCCTTTATTGCAAAGATAAAGCAGA
TTCCTCTAGTACAAGTGGGGAACAAAATAACGTGGAAAAGAGCTGTCCTGACAGCCCAC
TCACTAATGCCTATGACGAACGCAGTGACGACCACAAAACAATGGAGGGCCCCAGGCAC
GTGAAGACGCGTCTGCCGACAGTGGGTCTCGGACAACAGACACCACGCACTCAGCAAGT
GGATGAAATAATTCCTCTGCTGACGTAAGGGATGACGATCAATCCCACTATCCCAAGACC
CTTCACTTCTATATAACCGAAGTTGCTTCATTTGGAGA**A**GGCATCTCGAAATCTCAACAC
AACTCGAGCTCTCCCTTCTCTCTTCTTTATCTCTCTAAATGTGTGAGTAGA

>D2 (716 bp; 5'→3')
TTCGTCCACAGACATCAACATCTTATCGTCCTTTGAAGACAAGATAATAATGTTGAAGAT
AAGAGTGGGAGCCACCACTAAAACATTGCTTTGTCAAAAGCTAAAAAAGATGATGCCCG
ACAGCCACTTGTGTGAAGCATGAGAAGCCGGTCCCTCCACTAAGAAAATTAGTGAAGCA
TCTTCCAGTGGTCCCTCCACTCACAGCTCAATCAGTGAGCAACAGGACGAAGGAAATGA
CGTAAGCCATGACGTCTAATCCAGCTGGCTTGTGGGGACCAGACAAAAAAGGAATGGTG
CAGAATTGTTAGGCGCACCTACCAAAAGCATCTTTGCCTTTATTGCAAAGATAAAGCAGA
TTCCTCTAGTACAAGTGGGGAACAAAATAACGTGGAAAAGAGCTGTCCTGACAGCCCAC
TCACTAATGCGTATGACGAACGCAGTGACGATCACAAAACAATGGAGGGCCCCAGACAC
GTGAAGACGCGTCTGCCGACAGTGGGTCTCGGACAACAGACACCACGCACTCAGCAAGT
GGATGAAATAATTCATCTGCTGACGTAAGGGATGACGATCAATCCCACTATCCCAAGAC
CCTTCACTTCTATATAAGTGAAGTTGCTTCATTTGGAGA**A**GGCATCTCGAAATCTCAACA
CAACTCGAGCTCTCCCTTCTCTCTTCTTTATCTCTCTAAATGTGTGAGTAGA

>D3 (515 bp; 5'→3')
TTCGTCCACAGACATCAACATCTTATCGTCCTTTGAAGATAAGATAATAATGTTGAAGAT
AAGAGTGGGAGCCACCACTAAAACATTGCTTTGTCAAAAGCTAAAAAAGATGATGCCCG
ACAGCCACTTGTGTGAAGCATGTGAAGCCGGTCCCTCCACTAAGAAAATTAGTGTAGCAT
CTTCCAGTGGTCCCTCCACTCACGGCTCAATCAGTGAGCAACAGGACGAAGGAAATGAC
GTAAGCCATGACGTCTAATCCCAATGGAGGGCCCCAGATACGTGAAGACGCGTCTGCCG
ACAGTGGGTCTCGGACAACAGACACCACGCACTCAGCAAGTGGATGAAATAATTCATCT
GCTGACGTAAGGGATGACGATCAATCCCACTATCCCAAGACCCTTCACTTCTATATAAGT
GAAGTTGCTTCATTTGGAGA**A**GGCATCTCGAAATCTCAACACAACTCGAGCTCTCCCTTC
TCTCTTCTTTATCTCTCTAAATGTGTGAGTAGA
>D4 (385 bp; 5'→3')

TTCGTCCACAGACATCAACATCTTATCGTCCTTTGAAGATAAGATAATAATGTTGAAGAT
AAGAGTGGGAGCCACCACTAAAACATTGCTTTGTCAAAAGCTAAAAAAGATGATGCCCG
ACAGCCACTTGTGTGAAGCATGTGAAGCCGGTCCCTCCACTAAGAAAATTAGTGAAGCA
TCTTCCAGTGGTCCCTCCACTCACAGCTCAATCAGTGAGCAACAGGACGAAGGAAATGA
CGTAAGGGATGACGATCAATCCCACTATCCCAAGACCCTTCACTTCTATATAAGTGAAGT

TGCTTCATTTGGAGA**A**GGCATCTCGAAATCTCAACACAACTCGAGCTCTCCCTTCTCTCTT
CTTTATCTCTCTAAATGTGTGAGTAGA

>**D5 (**899 bp; 5’→3’)
AGCTGGCTTGTGGGGACCAGACAAAAAAGGAATGGTGCAGAATTGTTAGGCGCACCTAC
CAAAAGCATCTTTGCCTTTATTGCAAAGATAAAGCAGATTCCTCTAGTACAAGTGGGGAA
CAAAATAACGTGGAAAAGAGCTGTCCTGACAGCCCACTCACTAATGCGTATGACGAACG
CAGTGACGACCACAAAATTCGTCCACAGACATCAACATCTTATCGTCCTTTGAAGATAAG
ATAATAATGTTGAAGATAAGAGTGGGAGCCACCACTAAAACATTGCTTTGTCAAAAGCT
AAAAAAGATGATGCCCGACAGCCACTTGTGTGAAGCATGTGAAGCCGGTCCCTCCACTA
AGAAAATTAGTGAAGCATCTTCCAGTGGTCCCTCCACTCACAGCTCAATCAGTGAGCAAC
AGGACGAAGGAAATGACGTAAGCCATGACGTCTAATCCAGCTGGCTTGTGGGGACCAGA
CAAAAAAGGAATGGTGCAGAATTGTTAGGCGCACCTACCAAAAGCATCTTTGCCTTTATT
GCAAAGATAAAGCAGATTCCTCTAGTACAAGTGGGGAACAAAATAACGTGGAAAAGAG
CTGTCCTGACAGCCCACTCACTAATGCGTATGACGAACGCAGTGACGACCACAAAACAA
TGGAGGGCCCCAGACACGTGAAGACGCGTCTGCCGACAGTGGGTCTCGGACAACAGACA
CCACGCACTCAGCAAGTGGATGAAATAATTCATCTGCTGACGTAAGGGATGACGATCAA
TCCCACTATCCCAAGACCCTTCACTTCTATATAAGTGAAGTTGCTTCATTTGGAGA**A**GGC
ATCTCGAAATCTCAACACAACTCGAGCTCTCCCTTCTCTCTTCTTTATCTCTCTAAATGTG
TGAGTAGA

>**D6** (470 bp; 5’→3’)
TTCGTCCACAGACATCAACATCTTATCGTCCTTTGAAGATAAGATAATAATGTTGAAGAT
AAGAGTGGGAGCCACCACTAAAACATTGCTTTGTCAAAAGCTAAAAAAGATGATGCCCG
ACAGCCACTTGTGTGAAGCATGTGAAGCCGGTCCCTCCACTCACAGCTCAATCAGTGAGC
AACAGGACGAAGGAAATGACGTAAGCCATGACGTCTAATCCCAATGGAGGGCCCCAGAC
ACGTGAAGACGCGTCTGCCGACAGTGGGTCTCGGACAACAGACACCACGCACTCAGCAA
GTGGATGAAATAATTCATCTGCTGACGTAAGGGATGACGATCAATCCCACTATCCCAAG
ACCCTTCACTTCTATATAAGTGAAGTTGCTTCATTTGGAGA**A**GGCATCTCGAAATCTCAA
CACAACTCGAGCTCTCCCTTCTCTCTTCTTTATCTCTCTAAATGTGTGAGTAGA

>**D7** (384 bp; 5’→3’)
TTCGTCCACAGACATCAACATCTTATCGTCCTTTGAAGATAAGATAATAATGTTGAAGAT
AAGAGTGGGAGCCACCACTAAAACATTGCTTTGTCAAAAGCTAAAAAAGATGATGCCCG
ACAGCCACTTGTGTGAAGCATGTGAAGCCGGTCCCTCCACTAAGAAAATTAGTGAAGCA
TCTTCCAGTGGTCCCTCCACTCACAGCTCAATCAGTGAGCAACAGGACGAAGGAAATGA
CGTAAGCCATGACGTCTAATCCCCTATCCCAAGACCCTTCACTTCTATATAAGTGAAGTT
GCTTCATTTGGAGA**A**GGCATCTCGAAATCTCAACACAACTCGAGCTCTCCCTTCTCTCTTC
TTTATCTCTCTAAATGTGTGAGTAGA

>**D8** (198 bp; 5’→3’)
TTCGTCCACAGACATCAACATCTTATCGTCCTTTGAAGATAAGATAATAATGTTGAAGAT
AAGAGTGGGAGCCACCACTAAAACATTGCTTTGTCAAAAGCTAAAAAAGATGATGCCCG
ACAGCCACTTGTGTGAAGCATGTGAAGCCGGTCCCTCCCTCCCTTCTCTCTTCTTTATCTC
TCTAAATGTGTGAGTAGA

Sequence of oligo block shuffled promoter Sh6, where TATA Box has been labeled in red and TSS as bold yellow highlighted.

>**Sh6** (508 bp; 5'→3')

GTGGCCCGCAAGGTGAGGGCTTGTGGGGACAGACAAAAAAGGAATGGTGCAGAATTGTT
AAGTTGAAGATAAGATAATAATGTTGAAGATAAGAGTGGGAGCCACCACAGTGTGAAGC
ATGTGAAGCCGGTCCCTCCACTAAGAAAATTAGTGAAGCGCACCTACCAAAAGCATCTTT
GCCTTTATTGCAAAGATAAAGAGTTGAAGATAAGATAATAATGTTGAAGATAAGAGTGG
GAGCCACCACAGTTCGTCCACAGACATCAACATCTTATCGTCCTAGGAGCAACAGGACG
AAGGAAATGACGTAAGCCATGACGTCTAATCCAGTTCGTCCACAGACATCAACATCTTAT
CGTCCTAGCGCACCTACCAAAAGCATCTTTGCCTTTATTGCAAAGATAAAGAGCTGCTGA
CGTAAGGGATGACGATCAATCCCACTATCCCAAGACCGGTCTAGAAGACCCTTCACTTCT
ATATAAGTGAAGTTGCTTCATTTGGAGA**A**GGC

Supplementary Data 2: Sequences of primers and oligonucleotides used.

1) Primer used for hybrid promoter development

**Serial Number**	**Promoter fragment**	**Primer sequence (F= Forward, R= Reverse; 5'→3')**
1	H12	**H12 F:** GCCCCGGAATTCGTCGACTGGTAGACTATGAAACAC<br>**HRLV R:** ATGCAGAAGCTTTCTACTCACACATTTA
2	H17	**H17 F:** GGGCGAATTCGTCGACCAATGGAGGGCCCCAGACA<br>**HRLV R**
3	H17uas	**H17 F**<br>**H17uas R:** ATGCAGAAGCTTCCCGGGGAAGTGAAGGGTCTTG
4	Muas	**Muas F:** GCTAGAATTCGTCGACTTCGTCCACAGACATCAACAT<br>**Muas R:** ATCTAAAGCTTCCCGGGGGATTAGACGTCATGGCT
5	Fuas	**Fuas F:** ACTGCAGAATTCGTCGACAGCTGGCTTGTGGGGA<br>**Fuas R:** ACTAAAGCTTCCCGGGTTTTGTGGTCGTCACTGCG

2) Oligonucleotides used for oligo block shuffling.

**Serial Number**	**Promoter fragment**	**Sequence (F= Forward, R= Reverse; 5'→3')**
1	H17 min	**Sense**:<br>CTAGAAGACCCTTCACTTCTATATAAGTGAAGTTGCTTCATTTGGA<br>GAAGGC<br>**Anti-Sense**:<br>AGCTTGCCTTCTCCAAATGAAGCAACTTCACTTATATAGAAGTGA<br>AGGGTCTT
2	H17-1	**Sense**:<br>AGCAATGGAGGGCCCCAGACACGTGAAGACGCGTCTGCCGACAG<br>T<br>**Anti-Sense**:<br>CTACTGTCGGCAGACGCGTCTTCACGTGTCTGGGGCCCTCCATTG
3	H17-2	**Sense**:<br>AGGGTCTCGGACAACAGACACCACGCACTCAGCAAGTGGATGAA<br>A<br>**Anti-Sense**:<br>CTTTTCATCCACTTGCTGAGTGCGTGGTGTCTGTTGTCCGAGACC

4	H17-3	**Sense**:<br>AGCTGCTGACGTAAGGGATGACGATCAATCCCACTATCCCAAGACC<br>**Anti-Sense**:<br>CTGGTCTTGGGATAGTGGGATTGATCGTCATCCCTTACGTCAGCAG
5	Fuas-1	**Sense**:<br>AGGGCTTGTGGGGACCAGACAAAAAAGGAATGGTGCAGAATTGTTA<br>**Anti-Sense**:<br>CTTAACAATTCTGCACCATTCCTTTTTTGTCTGGTCCCCACAAGCC
6	Fuas-2	**Sense**:<br>AGCGCACCTACCAAAAGCATCTTTGCCTTTATTGCAAAGATAAAG<br>**Anti-Sense**:<br>CTCTTTATCTTTGCAATAAAGGCAAAGATGCTTTTGGTAGGTGCG
7	Fuas-3	**Sense**:<br>AGATTCCTCTAGTACAAGTGGGGAACAAAATAACGTGGAAAAG<br>**Anti-Sense**:<br>CTCTTTTCCACGTTATTTTGTTCCCCACTTGTACTAGAGGAAT
8	Fuas-4	**Sense**:<br>AGCCACTCACTAATGCGTATGACGAACGCAGTGACGACCACAAAA<br>**Anti-Sense**:<br>CTTTTTGTGGTCGTCACTGCGTTCGTCATACGCATTAGTGAGTGG
9	Muas-1	**Sense**:<br>AGTTCGTCCACAGACATCAACATCTTATCGTCCT<br>**Anti-Sense**:<br>CTAGGACGATAAGATGTTGATGTCTGTGGACGAA
10	Muas-2	**Sense**:<br>AGTTGAAGATAAGATAATAATGTTGAAGATAAGAGTGGGAGCCACCAC<br>**Anti-Sense**:<br>CTGTGGTGGCTCCCACTCTTATCTTCAACATTATTATCTTATCTTCAA
11	Muas-3	**Sense**:<br>AGATTGCTTTGTCAAAAGCTAAAAAAGATGATGCCCGACAGCCA

		**Anti-Sense**: CTTGGCTGTCGGGCATCATCTTTTTTAGCTTTTGACAAAGCAAT
12	Muas-4	**Sense**: AGTGTGAAGCATGTGAAGCCGGTCCCTCCACTAAGAAAATTAGTGA<br>**Anti-Sense**: CTTCACTAATTTTCTTAGTGGAGGGACCGGCTTCACATGCTTCACA
13	Muas-5	**Sense**: AGGAGCAACAGGACGAAGGAAATGACGTAAGCCATGACGTCTAATCC<br>**Anti-Sense**: CTGGATTAGACGTCATGGCTTACGTCATTTCCTTCGTCCTGTTGCTC
14	5’ Adapter	**Sense:** AATTCGTGGCCCGCAAGGTG<br>**Anti-Sense**: CTCACCTTGCGGGCCACG
15	3’ Adapter	**Sense:** AGCGGTCTAGACAGATT<br>**Anti-Sense**: CTAGAATCTGTCTAGACCG

3) Primers used for as-1 elements site-directed mutagenesis

**Serial Number**	**Promoter Mutant**	**Primer sequence (F= Forward, R= Reverse; 5’→3’)**
1	M	**F:** ACGAAGGAAACAGCGTAAGCCATTCGCTCTAATCCA<br>**R:** TGGATTAGAGCGAATGGCTTACGCTGTTTCCTTCGT
2	F	**F:** CTAATGCGTAGTGCGAACGCAGTTCAGACCACAAAA<br>**R:** TTTTGTGGTCTGAACTGCGTTCGCACTACGCATTAG
3	H	**F:** TAATTCATCTGCTTGCGTAAGGGATTCAGATCAATCCCA<br>**R:** GTGGGATTGATCTGAATCCCTTACGCAAGCAGATGAATTA

4) Primer sequences of 35S, GUS, GFP, nptII, rbcSE9, RT-GUS and RT-18S

**Serial Number**	**Name**	**Primer sequence (F= Forward, R= Reverse; 5’→3’)**
1	35S	**35S F:** ACTGAATTCGAGAAGATTAGCCTTTTCAATTT<br>**35S R:** GCTGAAATCACCAGTCTCTCTCTAAGCTTAGT
2	GUS	**GUS F:** GATCGCGAAAACTGTGGAAT

		**GUS R**: TAATGAGTGACCGCATCGAA
3	GFP	**GFP F:** ATGGTGAGCAAGGGCGAG<br><br>**GFP R**: TTACTTGTACAGCTCGTCC
5	rbcSE9	**rbsSE9 F**: GCGTCCGGATCCGCTTTCGTTCGTATCATCGGTTTC<br><br>**rbcSE9 R**: ATGTAGTCTAGATGATGCATGTTGTCAATCAATTGG
6	nptII	**nptII F**: ATGGCAATTACCTTATCCGCAACT<br>**nptII R**: TCAGAAGAACTCGTCAAGAAGGCG
7	RT-GUS	**RT-GUS-F:** GCTGTGCCTGAACCGTTATT<br><br>**RT-GUS-R:** CCAGCCATGCACACTGATAC
8	RT-Nt18S	**RT-18S-F:** GCAAATTACCCAATCCTGAC<br><br>**RT-18S-R:** CTATTGGAGCTGGAATTACC
9	RT-AtActin	**RT-Actin-F:** TTACCCGATGGGCAAGTC<br><br>**RT-Actin-R:** GCTCATACGGTCAGCGATAC

Supplementary Data 3: Gel image showing the restriction digestion products of rationally designed hybrid constructs in vectors (a) pUC119 and (b) pKYLXGUS using EcoRI and HindIII enzymes.

a

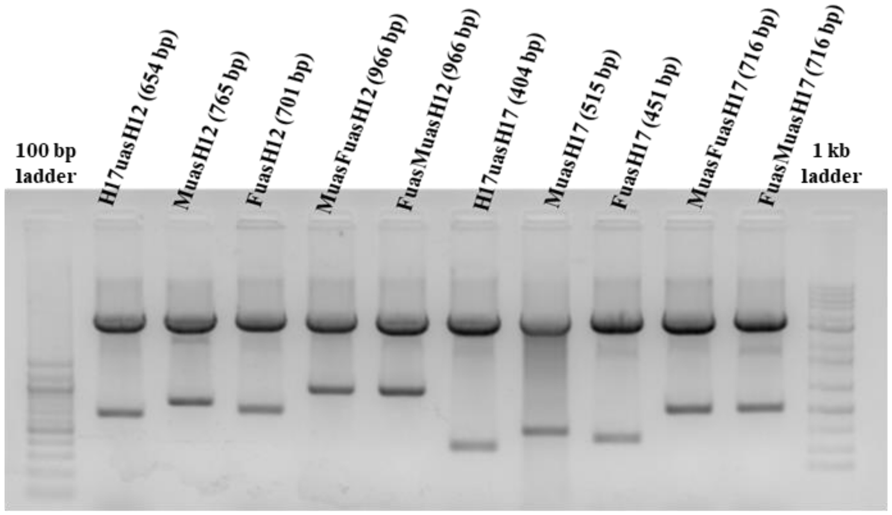

b

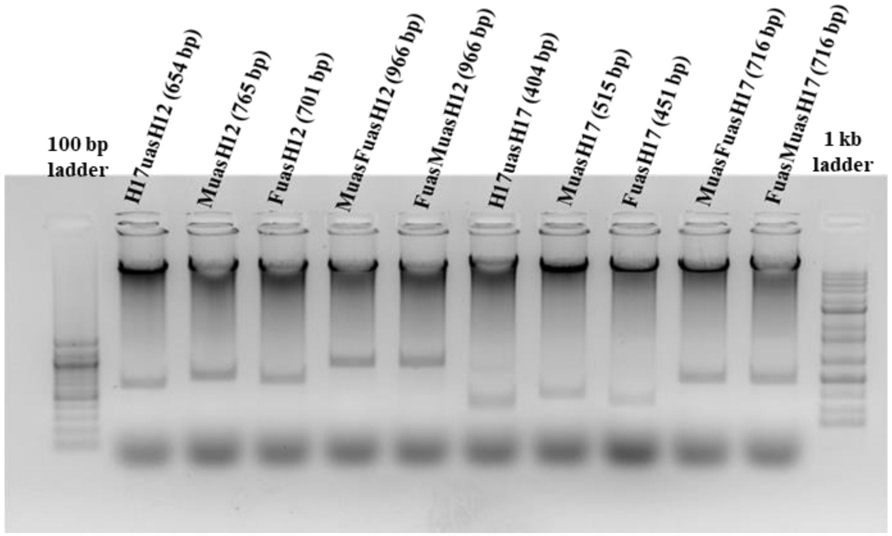

Supplementary Data 4: Cis-regulatory elements present in MFH17 promoter. The transcription start site (TSS) has been annotated as +1 and all the cis-regulatory elements has been labelled and coordinated, accordingly. The CCCGAC (inside square boxes) are the junction between Muas, Fuas and H17 promoter fragments, formed during fragment hybridization.

5’→3’

GATA motif (-597) GATA motif (-578) S Box (-567) CAAT Box (-555)

-643 TTCGTCCACAGACATCAACATCTTATCGTCCTTTGAAGATAAGATAATAATGTTGAAGATAAGAGTGGGAGCCACCACTAAAACATTGCTTTGTCAAAAGC

TCA element (-530) CAAT Box (-515) MYC (-500)

-542 TAAAAAAGATGATGCCCGACAGCCACTTGTGTGAAGCATGTGAAGCCGGTCCCTCCACTAAGAAAATTAGTGAAGCATCTTCCAGTGGTCCCTCCACTCA

MYB (-420) as-1 (-404) as-1 (-392)

-442 CAGCTCAATCAGTGAGCAACAGGACGAAGGAAATGACGTAAGCCATGACGTCTAATCCCCGACAGCTGGCTTGTGGGGACCAGACAAAAAAGGAAT

CAAT Box (-333) CAAT Box (-296) ABRE and G Box (-247)

-345 GGTGCAGAATTGTTAGGCGCACCTACCAAAAGCATCTTTGCCTTTATTGCAAAGATAAAGCAGATTCCTCTAGTACAAGTGGGGAACAAAATAACGTGG

as-1 (-204) as-1 (-192) CAAT Box (-173) ABRE and G Box (-154)

-246 AAAAGAGCTGTCCTGACAGCCCACTCACTAATGCGTATGACGAACGCAGTGACGACCACAAAACCCGACCAATGGAGGGCCCCAGACACGTGAAGAC

DRE (-137) MYB and GARE motif (-117) as-1 (-73) as-1 (-61) CAAT Box (-55)

-149 GCGTCTGCCGACAGTGGGTCTCGGACAACAGACACCACGCACTCAGCAAGTGGATGAAATAATTCATCTGCTGACGTAAGGGATGACGATCAATCCCAC

TATC-Box (-43) TATA Box (-22) TSS (+1)

-50 TATCCCAAGACCCTTCACTTCTATATAAGTGAAGTTGCTTCATTTGGAGAAGGCATCTCGAAATCTCAACACAACTCGAGCTCTCCCTTCTCTCTTCTTTATC

+53 TCTCTAAATGTGTGAGTAGA +73

Supplementary Data 5: Segregation analysis data of MFH17 transgenic *Nicotiana tabacum* and *Arabidopsis thaliana* T_1 generation plant lines

**Transgenic Plant lines**	**Total seeds germinated**	**Kan^R: Kan^S ratio**	**Phenotype of plant**	***Chi*-square value**
*Nt*MFH17-L1	97	80:17	Good growth	10.903037
***Nt*MFH17-L2**	**92**	**58:34**	**Good growth**	**0.566051**
*Nt*MFH17-L3	101	80:21	Stunted growth	7.08049
*Nt*MFH17-L4	90	67:23	Good growth	2.45
*Nt*MFH17-L5	86	66:20	Good growth	3.924645
*Nt*MFH17-L6	110	75:35	Stunted growth	0.112739
*Nt*MFH17-L7	105	80:25	Good growth	4.285714
*Nt*MFH17-L8	97	71:26	Good growth	1.858962
*Nt*MFH17-L9	85	61:24	Good growth	0.992647
*Nt*MFH17-L10	80	59:21	Good growth	1.802228
*At*MFH17-L1	135	101:34	Stunted growth	4.033333
*At*MFH17-L2	121	89:32	Good growth	2.580686
*At*MFH17-L3	153	112:41	Good growth	2.941176
***At*MFH17-L4**	**145**	**93:52**	**Good growth**	**0.418015**
*At*MFH17-L5	134	100:34	Stunted growth	3.816406
*At*MFH17-L6	120	85:35	Good growth	0.9375
*At*MFH17-L7	155	95:60	Stunted growth	2.019486
*At*MFH17-L8	102	72:30	Stunted growth	0.705882
*At*MFH17-L9	98	74:24	Good growth	3.444027
*At*MFH17-L10	126	106:20	Good growth	17.285714

Supplementary Data 6: Gene integration PCR of MFH17 promoter containing transgenic *Nicotiana tabacum* and *Arabidopsis thaliana* T_2 generation plants

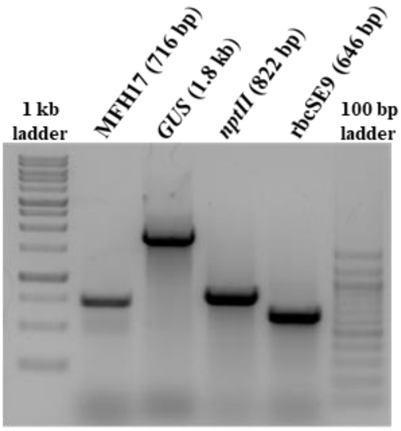

Nicotiana tabacum, Line 2

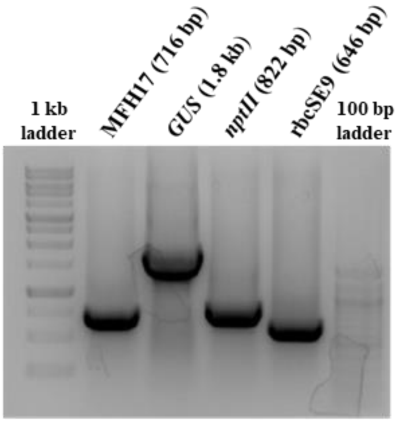

Arabidopsis thaliana, Line 4

Supplementary Data 7: Raw data of fluorometric GUS assays from important experiments where FSU 0', FSU 10', and FSU 20' represents the fluorescence measurements at 0, 10, and 20 minutes, respectively, expressed in Fluorescence Standard Units (FSU), ΔFSU1= 10'FSU-0'FSU, ΔFSU2= 20'FSU-10'FSU, Conc.= Concentration of total protein, A1= =ΔFSU1/(10 * Conc. * 25), A2= ΔFSU2/ (10 * Conc. * 25), GUS1= A1/ 7839.2, GUS2= A2/ 7839.2 and AVG= (GUS1+GUS2)/ 2.

Table 7.1. Raw data of MUG assay for the analysis of GUS activity in rationally designed hybrid promoters

Construct	0'FSU	10'FSU	20'FSU	ΔFSU1	ΔFSU2	Conc.	A1	A2	GUS1	GUS2	AVG
**Ctrl**	425	400	758	-25	358	0.0014	-71.4286	1022.857	-0.00911	0.13048	0.060684
**Ctrl**	785	458	859	-327	401	0.00758	-172.559	211.6095	-0.02201	0.026994	0.002491
**Ctrl**	652	754	801	102	47	0.004	102	47	0.013012	0.005996	0.009504
**35S**	747	71050	113635	70303	42585	0.008	35151.5	21292.5	4.484067	2.716157	3.600112
**35S**	893	68066	119878	67173	51812	0.0076	35354.21	27269.47	4.509926	3.478604	3.994265
**35S**	581	75904	113553	75323	37649	0.0085	35446.12	17717.18	4.52165	2.260075	3.390862
**2X35S**	927	129395	242086	128468	112691	0.01	51387.2	45076.4	6.555159	5.750128	6.152643
**2X35S**	806	107069	181748	106263	74679	0.009	47228	33190.67	6.024594	4.233935	5.129265
**2X35S**	1186	98246	170879	97060	72633	0.008	48530	36316.5	6.190683	4.632679	5.411681
**MuasH17**	981	102572	189832	101591	87260	0.0085	47807.53	41063.53	6.098521	5.23823	5.668376
**MuasH17**	841	132132	232629	131291	100497	0.01	52516.4	40198.8	6.699204	5.127921	5.913563
**MuasH17**	885	103829	190455	102944	86626	0.009	45752.89	38500.44	5.836423	4.911272	5.373848
**MuasFuasH17**	778	75766	135813	74988	60047	0.0045	66656	53375.11	8.502908	6.808745	7.655827
**MuasFuasH17**	732	102615	245618	101883	143003	0.009	45281.33	63556.89	5.77627	8.107573	6.941922
**MuasFuasH17**	832	135606	264031	134774	128425	0.01	53909.6	51370	6.876926	6.552965	6.714945
**FuasH17**	885	53520	95390	52635	41870	0.006	35090	27913.33	4.476222	3.560737	4.01848
**FuasH17**	1614	97867	204300	96253	106447	0.0098	39286.94	43447.76	5.011601	5.542371	5.276986
**FuasH17**	991	106021	204314	105030	98293	0.009	46680	43685.78	5.954689	5.572734	5.763712
**FuasMuasH17**	723	62250	140309	61527	78059	0.007	35158.29	44605.14	4.484933	5.690012	5.087472
**FuasMuasH17**	878	96255	241461	95377	145206	0.01	38150.8	58082.4	4.86667	7.409225	6.137948
**FuasMuasH17**	703	76449	154605	75746	78156	0.008	37873	39078	4.831233	4.984947	4.90809
**MuasH12**	315	30181	65711	29866	35530	0.003	39821.33	47373.33	5.07977	6.043134	5.561452
**MuasH12**	595	25635	60585	25040	34950	0.003	33386.67	46600	4.258938	5.944484	5.101711
**MuasH12**	458	30256	80452	29798	50196	0.0041	29071.22	48971.71	3.708442	6.247029	4.977735
**MuasFuasH12**	503	20145	60526	19642	40381	0.0025	31427.2	64609.6	4.008981	8.241861	6.125421
**MuasFuasH12**	507	21025	70526	20518	49501	0.003	27357.33	66001.33	3.489812	8.419397	5.954604
**MuasFuasH12**	515	59535	125487	59020	65952	0.008	29510	32976	3.764415	4.206552	3.985483
**FuasH12**	526	4508	81114	3982	76606	0.0035	4550.857	87549.71	0.580526	11.1682	5.87436
**FuasH12**	1055	97849	205606	96794	107757	0.01	38717.6	43102.8	4.938973	5.498367	5.21867
**FuasH12**	751	53644	101144	52893	47500	0.005	42314.4	38000	5.397796	4.847433	5.122615
**FuasMuasH12**	608	48595	98562	47987	49967	0.0045	42655.11	44415.11	5.441258	5.665771	5.553515
**FuasMuasH12**	704	47272	93505	46568	46233	0.005	37254.4	36986.4	4.752322	4.718135	4.735228
**FuasMuasH12**	609	70313	129150	69704	58837	0.006	46469.33	39224.67	5.927816	5.003657	5.465736
**H17**	299	51000	123245	50701	72245	0.008	25350.5	36122.5	3.233812	4.607932	3.920872
**H17**	759	58475	84707	57716	26232	0.006	38477.33	17488	4.908324	2.23084	3.569582
**H17**	385	39417	74489	39032	35072	0.005	31225.6	28057.6	3.983264	3.579141	3.781202

**H12**	884	71679	128063	70795	56384	0.0068	41644.12	33167.06	5.312292	4.230924	4.771608
**H12**	831	71723	123823	70892	52100	0.006	47261.33	34733.33	6.028846	4.430724	5.229785
**H12**	460	38940	72347	38480	33407	0.005	30784	26725.6	3.926931	3.409225	3.668078
**H17uasH17**	523	29605	57333	29082	27728	0.003	38776	36970.67	4.946423	4.716127	4.831275
**H17uasH17**	424	27713	54489	27289	26776	0.0035	31187.43	30601.14	3.978394	3.903605	3.941
**H17uasH17**	374	19889	41400	19515	21511	0.003	26020	28681.33	3.319216	3.658707	3.488961
**H17uasH12**	376	40256	80452	39880	40196	0.0038	41978.95	42311.58	5.355004	5.397436	5.37622
**H17uasH12**	778	37178	75357	36400	38179	0.004	36400	38179	4.643331	4.870267	4.756799
**H17uasH12**	485	42503	98052	42018	55549	0.005	33614.4	44439.2	4.287989	5.668844	4.978416

Table 7.2. Raw data of MUG assay for the analysis of GUS activity from transgenic *Nicotiana tabacum* seedlings

Construct	0'FSU	10'FSU	20'FSU	ΔFSU1	ΔFSU2	Conc.	A1	A2	GUS1	GUS2	AVG
**Ctrl**	485	785	801	300	16	0.029	41.37931	2.206897	0.005279	0.000282	0.00278
**Ctrl**	499	511	574	12	63	0.017	2.823529	14.82353	0.00036	0.001891	0.001126
**Ctrl**	485	572	512	87	-60	0.027	12.88889	-8.88889	0.001644	-0.00113	0.000255
**MFH17**	2807	203921	377198	201114	173277	0.012	67038	57759	8.551638	7.367971	7.959805
**MFH17**	1565	194319	419446	192754	225127	0.016	48188.5	56281.75	6.14712	7.179527	6.663323
**MFH17**	1746	325890	346995	324144	21105	0.01	129657.6	8442	16.53965	1.076896	8.808271
**35S**	1011	70175	153582	69164	83407	0.01	27665.6	33362.8	3.529136	4.255893	3.892515
**35S**	310	60525	158952	60215	98427	0.009	26762.22	43745.33	3.413897	5.580331	4.497114
**35S**	1323	148519	245632	147196	97113	0.011	53525.82	35313.82	6.827969	4.504773	5.666371

Table 7.3. Raw data of MUG assay for the analysis of GUS activity from transgenic *Arabidopsis thaliana* seedlings

Construct	0'FSU	10'FSU	20'FSU	ΔFSU1	ΔFSU2	Conc.	A1	A2	GUS1	GUS2	AVG
**Ctrl**	487	514	568	27	54	0.09	1.2	2.4	0.000153	0.000306	0.00023
**Ctrl**	451	565	599	114	34	0.015	30.4	9.066667	0.003878	0.001157	0.002517
**Ctrl**	456	520	585	64	65	0.025	10.24	10.4	0.001306	0.001327	0.001316
**MFH17**	1425	157466	226624	156041	69158	0.009	69351.56	30736.89	8.846764	3.920922	6.383843
**MFH17**	925	117435	244356	116510	126921	0.012	38836.67	42307	4.954162	5.396852	5.175507
**MFH17**	1075	116234	216756	115159	100522	0.01	46063.6	40208.8	5.876059	5.129197	5.502628
**35S**	850	105025	205262	104175	100237	0.015	27780	26729.87	3.543729	3.40977	3.476749
**35S**	760	90525	175895	89765	85370	0.012	29921.67	28456.67	3.816929	3.630047	3.723488
**35S**	1851	195659	276170	193808	80511	0.02	38761.6	16102.2	4.944586	2.054062	3.499324
